# DFT approach towards accurate prediction of ^1^H/^13^C NMR chemical shifts for dipterocarpol oxime†

**DOI:** 10.1039/d3ra04688e

**Published:** 2023-10-30

**Authors:** Phong Q. Le, Nhu Q. Nguyen, Thien T. Nguyen

**Affiliations:** a School of Biotechnology, International University, VNU HCM Quarter 6, Linh Trung Ward, Thu Duc City Ho Chi Minh City Vietnam; b Faculty of Pharmacy, College of Medicine and Pharmacy, Duy Tan University Da Nang 550000 Vietnam nguyentrongthien@duytan.edu.vn; c Institute of Research and Development, Duy Tan University Da Nang 550000 Vietnam

## Abstract

A computational NMR approach for accurate predicting the ^1^H/^13^C chemical shifts of triterpenoid oximes featuring the screening of 144 DFT methods was demonstrated. Efficiently synthesized dipterocarpol oxime was employed as a model compound. The six highest accurate methods from the screening generated root-mean-square-error (RMSE) values in the range of 0.84 ppm (0.55%) to 1.14 ppm (0.75%) for calculated ^13^C shifts. For ^1^H results, simple, economical 6-31G basis set unexpectedly outperformed other more expensive basic sets; and the couple of it with selected functionals provided high accuracy shifts (0.0617 ppm (1.49%) ≤ RMSE ≤ 0.0870 ppm (2.04%)). These computational results strongly supported the proton and carbon assignments of the oxime including the difficult ones of diastereotopic methyl groups, the methyl groups attached to an internal olefin, and diastereotopic α-protons.

## Abstract 2

A computational NMR approach for accurate predicting the ^1^H/^13^C chemical shifts of triterpenoid oximes featuring the screening of 144 DFT methods was demonstrated.

## Graphical abstract

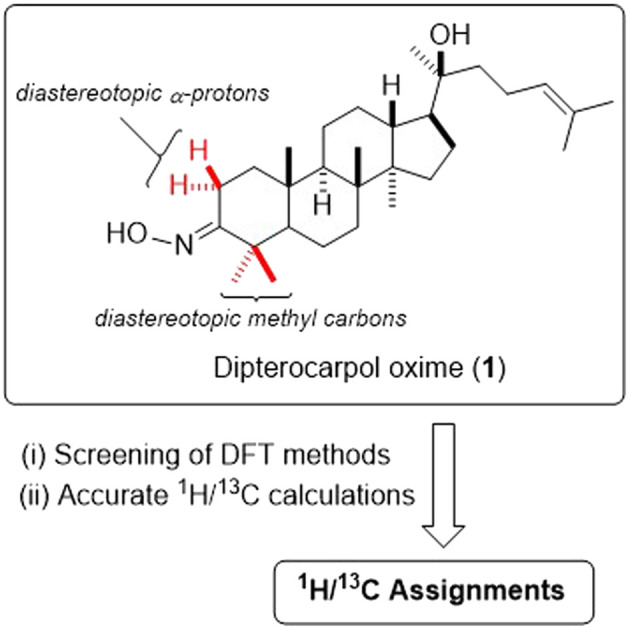

## Introduction

In the last two decades, the development of accurate ^1^H/^13^C NMR computation methods has been useful for the structure elucidation of complex organic molecules.^1^ Such methods provide the detailed information of local electron environment around the protons and carbon nuclei of the molecules, assist their full structure assignments, and contribute valuable insights into their conformations. Gauge-including atomic orbital (GIAO)/density functional theory (DFT) method^2,3,4^ has been generally accepted as a standard approach for computing shielding constants due to its high accuracy and reliability. It has effectively supported the structural assignments, validations, and corrections of natural products at affordable computational costs.^5^ The accuracy of calculated ^1^H/^13^C chemical shifts generally depends on geometry optimization, density functional methods, basis sets, solvent models, energy calculations, and NMR methods.^6,7^ Previous studies reported the impacts of different density functional methods and basis sets on ^1^H/^13^C NMR calculations using a benchmark of small-sized, organic structures (MW < 200 g mol^−1^),^8–12^ while large molecules have not been employed for such studies. This could be explained by the requirement of a skill set for experimental NMR analysis of large molecules and the high time cost for NMR computation. In addition, the report of Hehre *et al.* in 2019 showed that a single DFT method (ωB97XD/6-31G(d,p)) could not be applied for all large, flexible molecules.^13^ The screening of DFT methods, therefore, would be a necessary step for obtaining high accurate ^1^H/^13^C NMR chemical shift computation for such molecules.

**Fig. 1:**
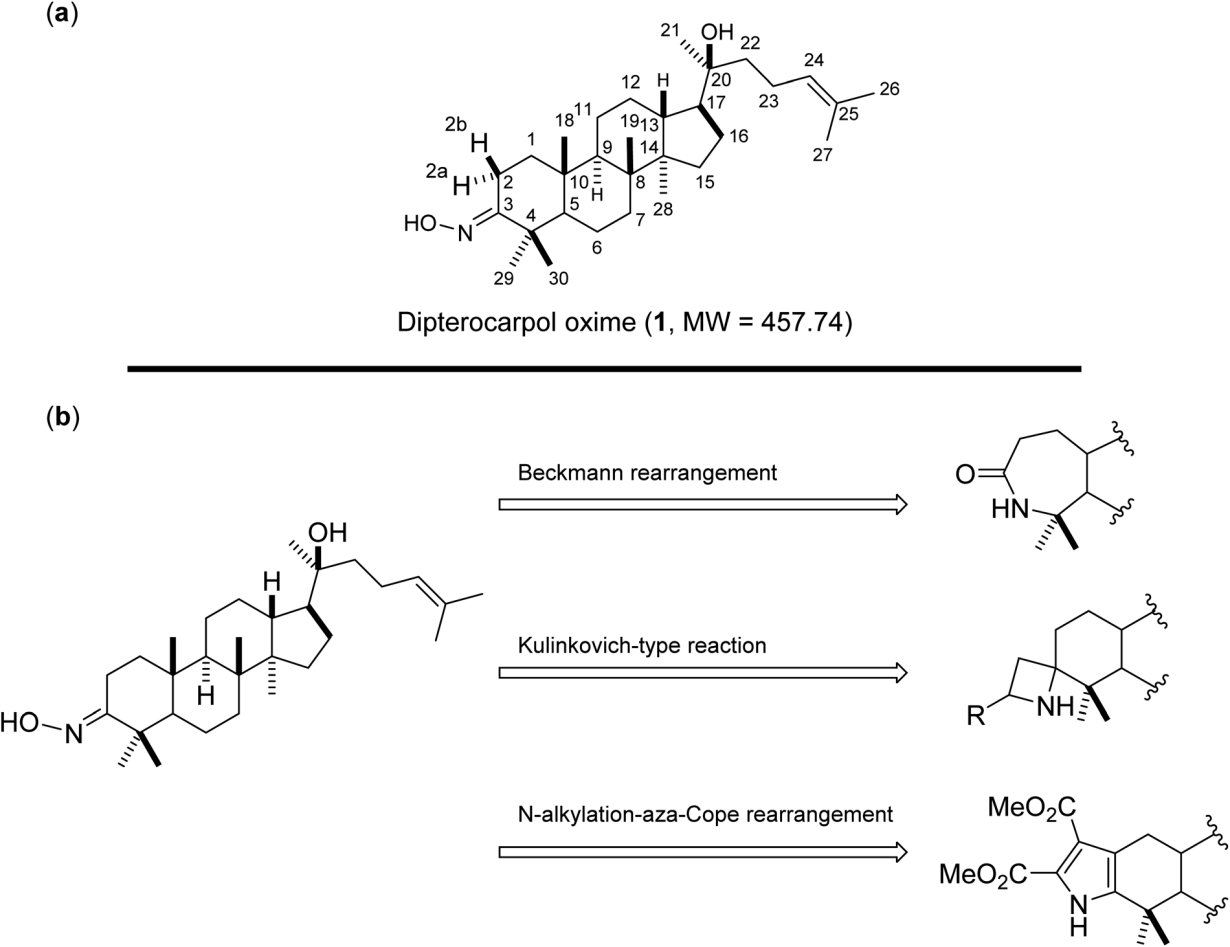
(a) Structures of oxime 1 and (b) synthetic potentials of the oxime functional group.

**Fig. 2:**
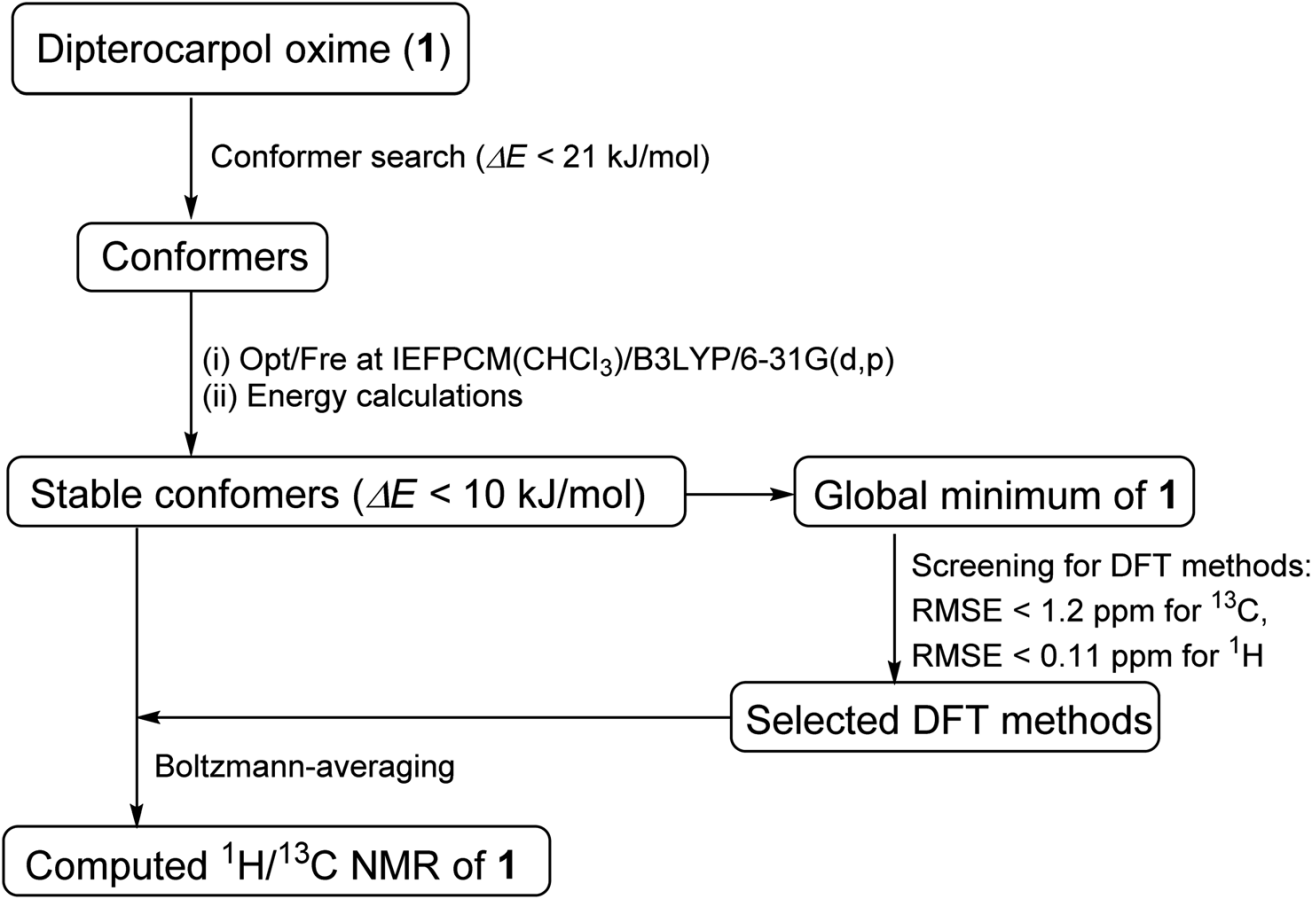
DFT approach toward accurate ^1^H/^13^C NMR chemical shift computation.

Oxime 1 (MW = 457.74) containing a dammarane skeleton (6/6/6/5-fused tetracyclic ring system)^14–16^ is a potential intermediate for the syntheses of many functional groups,^17^ such as lactams *via* Beckmann rearrangement,^18^ azetidines *via* Kulinkovich-type mechanism,^19^ and pyrroles *via N*-alkylation-aza-Cope rearrangement (Fig. 1b).^20^ The structure elucidation tasks of this triterpenoid derivative using 1D and 2D NMR, including the assignments of diastereotopic methyl groups (C29 and C30), the two methyl groups (C26 and C27) at the internal olefin, and diastereotopic α-protons (H2a and H2b) (Fig. 1a), are typically challenging due to similar coupling patterns and a broad overlapping of signals. Therefore, high accuracy ^1^H/^13^C calculations would significantly support the full structure interpretation of the oxime including those difficult assignments. Herein, we demonstrate the computational approach to accurate ^1^H/^13^C computation for oxime 1 (Fig. 2) featuring the screening of 144 combinations from 13 density functional methods and 11 basis sets and the utility of best performing combinations for ^1^H/^13^C chemical shift calculations.

## Computational methods

Conformer searches of oxime 1 were performed using Macromodel software. The MMFFs force field in gas phase was used due to the presence of a sp^2^-hybridized nitrogen in 1. 10^5^ steps of Mixed Torsional Low-Mode sampling algorithm with a maximum number of 1000 steps per rotable bond were used. A Polak–Ribier Conjugate Gradient minimization method with a maximum number of 2500 iterations and a convergence threshold of 0.05 were applied. A RMSE cutoff of 1.0 Å was used to reduce redundant conformations. Extended non-bonded cutoff distances with van der Waals cutoff of 8.0 Å and an electrostatic cutoff of 20.0 Å were applied. All local minima within 21 kJ mol^−1^ of the global minimum were saved. These conformers were optimized at the level IEFPCM(CHCl_3_)/B3LYP/6-31G(d,p) of theory using Gaussian09.^21^ Subsequent frequency calculations ensured that potential energy surface (PES) local minima were attained. Single-point energies were re-calculated for optimized geometries at the same level of theory with the grid size of ultrafine. Cartesian coordinates of these optimized geometries were given in the ESI.†

For the screenings of functional and basis set performances, the global minimum was employed. The following 13 functionals and 11 basis sets were chosen for this investigation due to their common uses for ^1^H/^13^C calculations.

### Functionals

B3LYP (Becke's 3-parameter hybrid functional using B exchange and LYP correlation),^22^ B3PW91 (Perdew and Wang's 1991 gradient-corrected correlation functional),^23^ B97D (Grimme's functional including dispersion),^24^ BPV86 (a Becke88 exchange and Perdew's 1986 functional),^25^ CAM-B3LYP (Handy and co-workers’ long-range corrected version of B3LYP using the Coulomb-attenuating method),^26^ HCTH (Hamprecht-Cohen-Tozer-Handy GGA functional),^27^ HSEH1PBE (The exchange part of the screened Coulomb potential of Heyd, Scuseria, and Ernzerhof),^28^ LSDA (Local spin-density approximation),^29^ M06-2X (a high-nonlocality functional with double the amount of nonlocal exchange),^30^ mPW1PW91 (mPW exchange and PW91 correlation),^31^ PBEPBE (The functional of Perdew, Burke, and Ernzerhof),^32^ TPSSTPSS (The exchange component of the Tao–Perdew–Staroverov–Scuseria),^33^ and ωB97XD (Head-Gordon and coworkers' dispersion corrected long-range corrected hybrid functional).^34^

### Basis sets

Pople's 6-31G, 6-31G(d,p), 6-31G(3d,p), 6-31G(d,3p), 6-31G(3d,3p), 6-31+G(d,p), 6-31++G(d,p), and 6-311G(d,p);^35^ Dunning's cc-pVDZ correlation consistent basis set;^36^ and DGDZVP and DGDZVP2.^37^

Single-point NMR GIAO calculations were carried out using the above density functional methods and basis sets. Integral equation formalism variant of the polarized continuum model (IEFPCM) was incorporated during NMR calculations.^38,39^ The GIAO NMR results were observed and extracted using GaussView05. Each optimized structure was used for computing the corresponding isotropic shielding constants (*σ*_cal_). To reduce the systematic error of the calculations, the linear regression analysis of calculated shielding constants *versus* the experimental ones (*δ*_exp_) (eqn (1)) were performed and the chemical shifts (*δ*_cal_) were computed according to eqn (2). The deviations between computed and experimental chemical shifts were given in the ESI.† For the ^1^H calculations, due to the overlapping proton signals in the experimental spectrum (SI), only assignable protons were considered for the calculations. An average of values of equivalents atoms was assumed. For example, single proton signals are experimentally observed for the methyl groups of 1 due to fast rotations around C–C bonds relative to the NMR measurement time scale. Computed results were evaluated using absolute deviations (|Δ*δ*|/ppm, eqn (3)); mean absolute error (MAE/ppm, eqn (4)); root mean squared error (RMSE/ppm, eqn (5)); and the coefficient of determination (*r*^2^). The smaller values of MAE and RMSE indicate smaller errors and the larger value of *r*^2^ means a stronger correlation between theoretical and experimental data. Error calculations and linear correlations were performed using Microsoft Excel 2013.

1 *σ*_cal_ = *aδ*_exp_ + *b*

2 *δ*_cal_ = (*σ*_cal_ − *b*)/*a*

3 |Δ*δ*| = |*δ*_scal_ − *δ*_exp_|

4
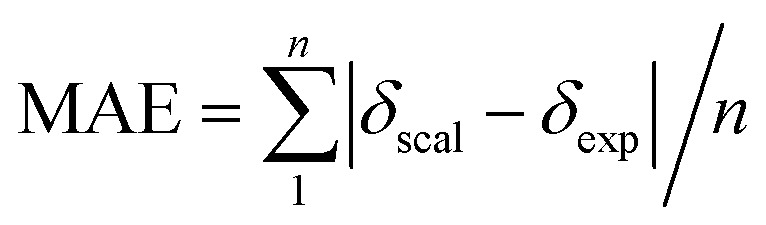
5
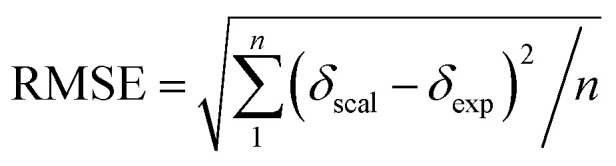


Major contributing conformers with a 10 kJ mol^−1^ energy window were used for NMR calculations. Boltzmann weighing average was calculated according to eqn (6), in which *σ*_*i*_ is the shielding constant, *E*_*i*_ is the relative energy of conformer *i*, *R* is the molar gas constant (8.3145 J K^−1^ mol^−1^), *T* is the temperature used for the calculation (298 K).

6 *σ*_cal_ = ∑_*i*_σ_*i*_e^−(*E*_*i*_/*RT*)^/∑_*i*_e^−(*E*_*i*_/*RT*)^

## Results and discussion

**Fig. 3:**
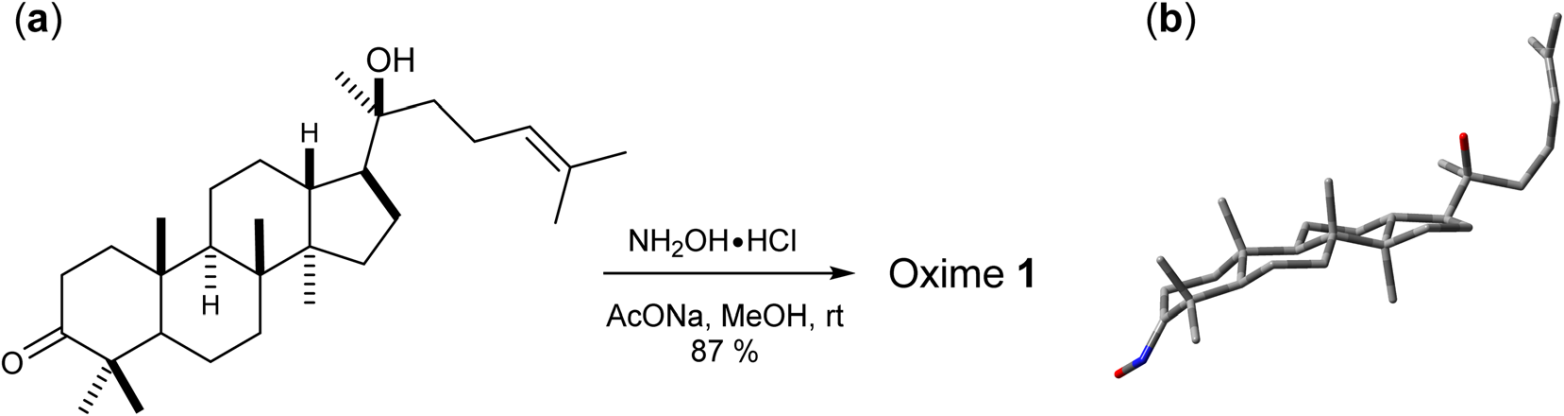
(a) Synthesis of oxime 1 and (b) its global minimum geometry optimized at IEFPCM(CHCl_3_)/B3LYP/6-31G(d,p).

Oxime 1 was efficiently prepared in 87% yield by the condensation of dipterocarpol (Fig. 3a), whose structure was confirmed by XRD analysis,^40^ with hydroxylamine. With a set of experimental ^1^H/^13^C NMR chemical shifts of 1 in hands, we proceeded with the computational study. Initially, the conformer searches of oxime 1 generated 38 conformers. After the optimizations and energy calculations, 15 conformers within the energy window of 10 kJ mol^−1^ from the global minimum were located. Fig. 3b showed the optimized geometry of the global minimum, which was adopted the chair conformation for all six-member rings including those containing a sp^2^-hybridized carbon.

### Screening of DFT methods

**Fig. 4:**
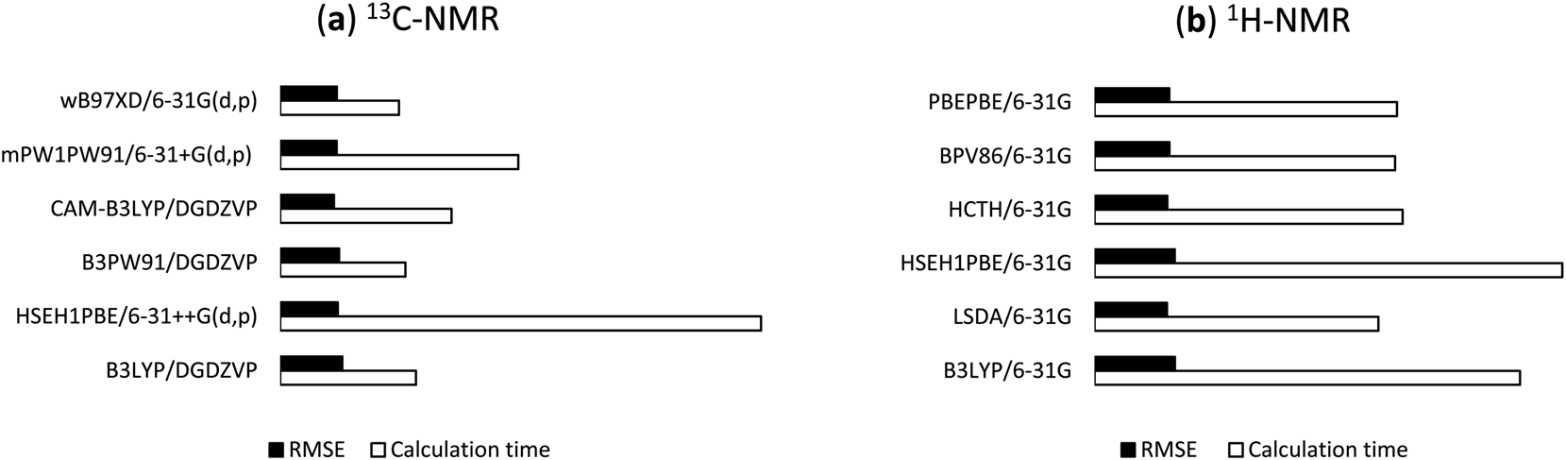
Relative RMSE values and NMR calculation times of the selected functionals for (a) ^13^C and (b) ^1^H.

144 DFT methods from the combinations of 13 functionals and 11 basis sets were tested for the ^1^H/^13^C NMR calculations of 1. For ^13^C calculations, B97D, BPV86, HCTH, LSDA, M06-2X, PBEPBE, and TPSSTPSS showed relatively less effective with *r*^2^ and RMSE values in the ranges of 0.9797 to 0.9984 and 1.41 ppm (0.93%) to 5.15 ppm (3.39%), respectively (Table S1, ESI†), even though the results would allow meaningful predictions of ^13^C chemical shifts. B3LYP, B3PW91, CAM-B3LYP, HSEH1PBE, mPW1PW91, and ωB97XD were more effective with *r*^2^ and RMSE values in the ranges of 0.9899 to 0.9992 and 1.03 ppm (0.68%) to 3.61 ppm (2.38%), respectively (Table 1). CAM-B3LYP was consistent in providing high accuracy results. Among tested basis sets, 6-31G(d,p) (entry 2), 6-31+G(d,p) (entry 3), DGDZVP (entry 10), and DGDZVP2 (entry 11) showed lowest errors (1.03 ppm (0.68%) ≤ RMSE ≤ 1.57 ppm (1.03%)). 6-31++G(d,p) (entry 4), 6-311G(d,p) (entry 5), cc-pVDZ (entry 9) had relatively good results (1.18 ppm (0.78%) ≤ RMSE ≤ 3.61 ppm (2.38%)) but these basis sets were computationally expensive. It was obvious that too many sets of polarity functions lead to lower accuracy and more computation costs (Table S3, ESI†). As showed in Table 1, the use of 6-31G(3d,3p) yielded much larger errors (1.96 ppm (1.29%) ≤ RMSE ≤ 3.61 ppm (2.38%)) in comparison to 6-31G(d,p). Overall, the screening provided top six combinations those with the highest accuracy of ^13^C chemical shifts, namely B3LYP/DGDZVP, B3PW91/DGDZVP, CAM-B3LYP/DGDZVP, HSEH1PBE/6-31++G(d,p), mPW1PW91/6-31G+(d,p), and ωB97XD/6-31G(d,p). The NMR calculation times of these combinations using a commercial computer with a Pentinum P6300 processor were showed in Table 3 and their relative RMSE values and times were provided in Fig. 4a.

**Table 1:** Screening density functional methods and basis sets for ^13^C calculations^a^

Entry	Basis set	B3LYP		HSEH1PBE		B3PW91		CAM-B3LYP		mPW1PW91		ωB97XD	
		*r* ^2^	RMSE	*r* ^2^	RMSE	*r* ^2^	RMSE	*r* ^2^	RMSE	*r* ^2^	RMSE	*r* ^2^	RMSE
1	6-31G	0.9981	1.54	0.9988	1.24	0.9985	1.37	0.9990	1.16	0.9988	1.24	0.9989	1.20
2	6-31G(d,p)	0.9983	1.50	0.9988	1.24	0.9985	1.37	0.9992	1.03	0.9989	1.20	0.9991	1.08
3	6-31+G(d,p)	0.9987	1.31	0.9990	1.12	0.9989	1.17	0.9991	1.06	0.9991	1.08	0.9987	1.27
4	6-31++G(d,p)	0.9988	1.25	0.9991	1.10	0.9990	1.13	0.9991	1.09	0.9990	1.13	0.9988	1.26
5	6-311G(d,p)	0.9983	1.49	0.9986	1.36	0.9984	1.43	0.9989	1.18	0.9987	1.30	0.9988	1.24
6	6-31G(d,3p)	0.9977	1.72	0.9985	1.37	0.9982	1.51	0.9989	1.20	0.9986	1.32	0.9988	1.21
7	6-31G(3d,p)	0.9930	3.01	0.9951	2.51	0.9939	7.72	0.9963	2.19	0.9950	2.54	0.9976	1.76
8	6-31G(3d,3p)	0.9899	3.61	0.9934	2.91	0.9915	3.31	0.9944	2.68	0.9930	3.00	0.9970	1.96
9	cc-pVDZ	0.9975	1.79	0.9983	1.48	0.9980	1.60	0.9989	1.20	0.9985	1.40	0.9989	1.19
10	DGDZVP	0.9989	1.19	0.9990	1.12	0.9990	1.13	0.9992	1.03	0.9990	1.14	0.9990	1.11
11	DGDZVP2	0.9981	1.57	0.9987	1.31	0.9984	1.43	0.9992	1.03	0.9988	1.26	0.9990	1.12

a Best performing combinations for each functional are in bold. RMSE values are in ppm.

For the predictions of ^1^H chemical shifts, only assignable protons from ^1^H and COSY NMR spectra were considered. Those are the protons of 8 methyl groups, an olefinic proton H24, diastereotopic protons H2a and H2b, and allylic protons H23, in which the COSY correlation signals of (H2a, H2b) and (H23, H24) were unambiguously assigned. The results of B3LYP, LSDA, HSEH1PBE, HCTH, BPV86, PBEPBE (Table 2) were better than those of the remaining density functional methods (Table S2, ESI†). Simple, economical 6-31G (entry 1) unexpectedly outperformed the other basis sets with *r*^2^ and RMSE values in the ranges of 0.9915 to 0.9936 and 0.098 ppm (2.30%) to 0.108 ppm (2.54%), respectively. Either adding diffusion or polarity function to the Pople's basis sets or using triple-zeta version (entries 2–8) significantly lowered the accuracy. The uses of cc-pVDZ, DGDZVP, and DGDZVP2 (entries 9–11) also generated larger errors. These results (Table 2) would strongly suggest that the screening for accurate ^1^H calculation is a necessary step for identifying suitable density functional methods and basis sets. The highest performing combinations, including B3LYP/6-31G, LSDA/6-31G, HSEH1PBE/6-31G, HCTH/6-31G, BPV86/6-31G, and PBEPBE/6-31G, were employed for the next step of ^1^H NMR calculations. The NMR calculation times of these combinations were showed in Table 3 and their relative RMSE values and times were provided in Fig. 4b.

**Table 2:** Screening density functional methods and basis sets for ^1^H calculations^a^

Entry	Basis set	B3LYP		LSDA		HSEH1PBE		HCTH		BPV86		PBEPBE	
		*r* ^2^	RMSE	*r* ^2^	RMSE	*r* ^2^	RMSE	*r* ^2^	RMSE	*r* ^2^	RMSE	*r* ^2^	RMSE
1	6-31G	0.9915	0.108	0.9931	0.098	0.9915	0.108	0.9930	0.098	0.9926	0.101	0.9926	0.101
2	6-31G(d,p)	0.9881	0.129	0.9897	0.120	0.9874	0.132	0.9886	0.126	0.9892	0.122	0.9889	0.124
3	6-31+G(d,p)	0.9882	0.128	0.9895	0.120	0.9881	0.128	0.9879	0.130	0.9892	0.122	0.9890	0.123
4	6-31++G(d,p)	0.9838	0.219	0.9855	0.142	0.9848	0.146	0.9813	0.161	0.9843	0.148	0.9841	0.149
5	6-311G(d,p)	0.9865	0.137	0.9882	0.128	0.9866	0.136	0.9875	0.132	0.9880	0.129	0.9875	0.132
6	6-31G(d,3p)	0.9864	0.137	0.9878	0.130	0.9863	0.138	0.9875	0.132	0.9879	0.130	0.9876	0.131
7	6-31G(3d,p)	0.9791	0.171	0.9786	0.173	0.9804	0.166	0.9796	0.169	0.9782	0.175	0.9782	0.175
8	6-31G(3d,3p)	0.9782	0.175	0.9775	0.178	0.9790	0.171	0.9756	0.185	0.9770	0.180	0.9769	0.180
9	cc-pVDZ	0.9856	0.141	0.9875	0.132	0.9851	0.144	0.9870	0.134	0.9876	0.131	0.9871	0.134
10	DGDZVP	0.9866	0.136	0.9890	0.124	0.9847	0.146	0.9871	0.134	0.9881	0.128	0.9877	0.130
11	DGDZVP2	0.9886	0.125	0.9907	0.113	0.9879	0.129	0.9901	0.117	0.9903	0.116	0.9900	0.118

a Best performing combinations for each functional are in bold. RMSE values are in ppm.

**Table 3:** NMR calculation times of the selected functionals^a^

	B3LYP	B3PW91	BPV86	CAM-B3LYP	HCTH	HSEH1PBE	LSDA	mPW1PW91	PBEPBE	ωB97XD
6-31G	0.57	0.57	0.41	0.71	0.42	0.63	0.38	0.58	0.41	0.71
6-31G(d,p)	1.94	1.87	1.02	2.25	0.99	1.87	0.95	1.88	0.99	2.27
6-31G(d,3p)	4.53	4.53	2.15	5.57	2.32	4.47	1.91	4.55	2.16	5.61
6-31G(3d,p)	9.03	8.66	3.55	10.65	3.53	9.19	3.18	8.54	3.54	11.59
6-31G(3d,3p)	16.63	16.65	6.54	22.36	6.61	17.20	5.87	17.20	6.45	21.97
6-31+G(d,p)	7.36	6.92	2.73	8.84	2.97	6.81	2.40	6.87	2.72	9.01
6-31++G(d,p)	11.18	12.56	4.46	14.84	4.41	11.20	4.68	11.28	4.45	15.21
6-311G(d,p)	4.02	4.14	1.87	4.69	1.90	4.15	1.69	4.07	1.87	4.89
cc-pVDZ	3.52	3.50	1.83	4.81	1.78	4.39	1.73	3.56	1.86	4.83
DGDZVP	2.59	2.39	1.33	3.27	1.32	2.81	1.32	2.41	2.92	3.25
DGDZVP2	4.92	4.60	2.27	6.15	2.27	5.09	2.10	4.33	2.27	6.03

a Time values are in hour.

### ^1^H/^13^C NMR calculations

**Fig. 5:**
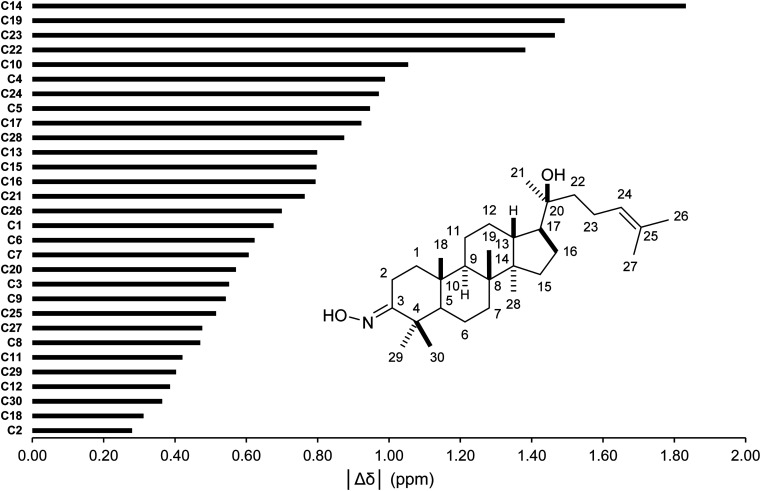
Averages of absolute deviations for ^13^C chemical shifts using selected combinations.

The conformer searches followed by optimizations and energy calculations resulted in 15 geometries within 10 kJ mol^−1^ energy window from the global minimum. Selected DFT methods and Boltzmann averaging were used for the ^1^H/^13^C NMR calculations. Table 4 showed the high accuracy results using six combinations for ^13^C shifts (0.9990 ≤ *r*^2^ ≤ 0.9994, 0.62 ppm (0.41%) ≤ MAE ≤ 0.89 ppm (0.59%), and 0.84 ppm (0.55%) ≤ RMSE ≤ 1.14 ppm (0.75%)). Among them, CAM-B3LYP/DGDZVP (entry 3) was the best performing methods with the lowest errors. Except HSEH1PBE/6-31G++(d,p), the other methods had |Δ*δ*_max_| below 3.00 ppm. These results would allow meaningful predictions for all carbon nuclei as showed in Fig. 5. All carbon atoms had the absolute deviation averages of the six calculation methods below 1.50 ppm, except C14 (|Δ*δ*| = 1.83 ppm). The high accuracy NMR calculations would strongly support the assignments of ambiguous carbon chemical shifts including those for diastereotopic methyl carbons (C29 and C30) and the two methyl carbons (C26 and C27) attached to the internal olefin. The calculation results showed that carbons C30 and C27 were more shielded than carbons C29 and C26, respectively. It should be noted that even with the careful analysis of 1D & 2D NMR the assignments of carbons C29 and C30 would be still challenging.

**Table 4:** ^13^C NMR calculations using selected combinations

Entry	Combination	*r* ^2^	MAE (ppm)	RMSE (ppm)	|Δ*δ*max| (ppm)
1	B3LYP/DGDZVP	0.9993	0.75	0.95	2.09
2	B3PW91/DGDZVP	0.9993	0.71	0.93	2.21
3	CAM-B3LYP/DGDZVP	0.9994	0.62	0.84	2.15
4	HSEH1PBE/6-31++G(d,p)	0.9991	0.89	1.09	3.17
5	mPW1PW91/6-31G+(d,p)	0.9990	0.89	1.14	2.73
6	ωB97XD/6-31G(d,p)	0.9994	0.72	0.90	2.08

**Fig. 6:**
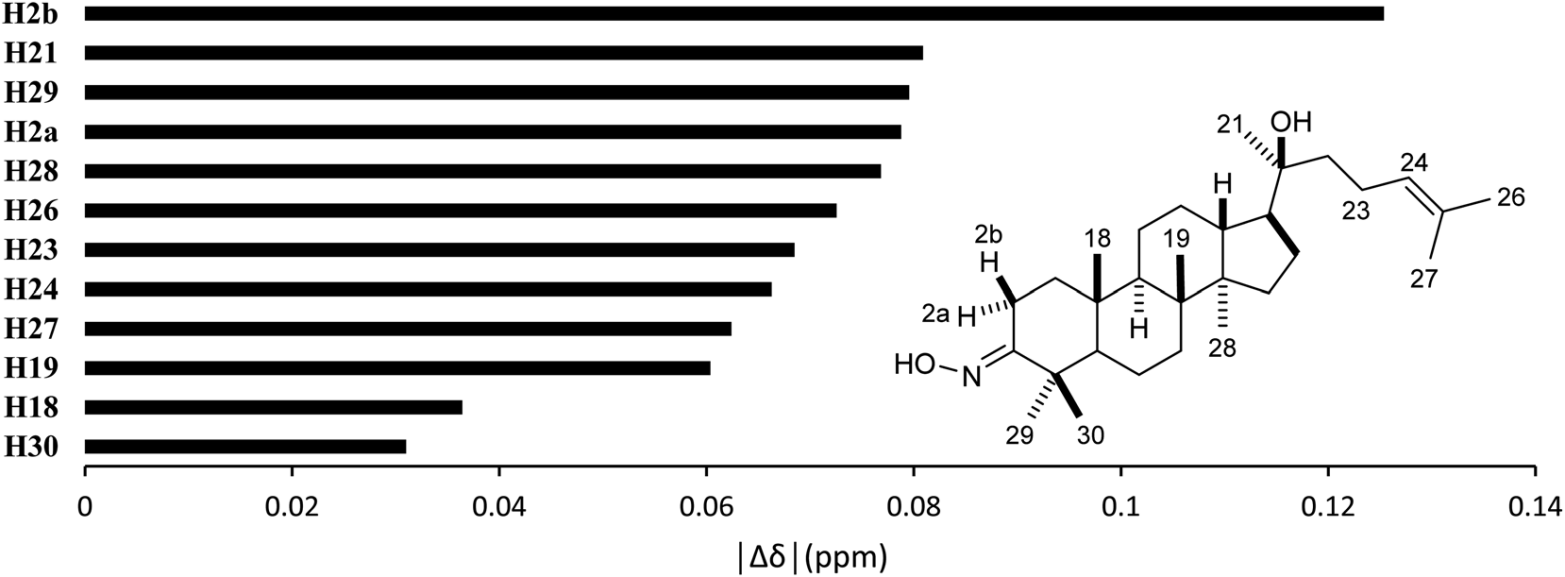
Averages of absolute deviations for ^1^H chemical shifts using selected combinations.

High accuracy ^1^H results (Table 5, 0.9945 ≤ *r*^2^ ≤ 0.9972, 0.0554 ppm (1.30%) ≤ MAE ≤ 0.0765 ppm (1.80%), and 0.0617 ppm (1.45%) ≤ RMSE ≤ 0.0870 ppm (2.04%)) were also obtained for the selected methods. The best performing combination was HSEH1PBE/6-31G (entry 3). Noticeable absolute deviation (|Δ*δ*| = 0.125 ppm) was observed for proton H2b (Fig. 6). This result can be explained by the electronic impact of the neighboring oxime group and the solvent effects, which is not sufficiently modelled by the selected DFT methods. The remaining protons had the errors below 0.100 ppm. The high accuracy calculations showed the average shift difference of 0.751 ppm between diastereotopic protons H2a and H2b, in which proton H2b were more shielded than proton H2a. This allowed the assignments of proton H2a (2.96 ppm) and proton H2b (2.27 ppm). The more crowded H2b-face of the six-membered ring could be resulted in its lower chemical shift.

**Table 5:** ^1^H NMR calculations using selected combinations

Entry	Combination	*r* ^2^	MAE (ppm)	RMSE (ppm)	|Δ*δ*max|(ppm)
1	B3LYP/6-31G	0.9969	0.0594	0.0647	0.107
2	LSDA/6-31G	0.9965	0.0614	0.0691	0.115
3	HSEH1PBE/6-31G	0.9972	0.0554	0.0617	0.109
4	HCTH/6-31G	0.9966	0.0646	0.0681	0.0940
5	BPV86/6-31G	0.9970	0.0606	0.0643	0.0940
6	PBEPBE/6-31G	0.9945	0.0765	0.0870	0.161

## Conclusion

The high accurate ^1^H/^13^C chemical shift calculations for dipterocarpol oxime 1 featuring the screening of 144 DFT methods from 13 density functional methods and 11 basis sets were achieved with the lowest RMSE values of 0.84 ppm (0.55%) and 0.0617 ppm (1.49%) for all 30 carbon atoms and 29 considered protons, respectively. B3LYP/DGDZVP, B3PW91/DGDZVP, CAM-B3LYP/DGDZVP, and ωB97XD/6-31G(d,p) were recommended for ^13^C calculations. Simple, economical 6-31G basis set coupled with HSEH1PBE, BPV86, and B3LYP unexpectedly provided highest accuracy ^1^H results. These results strongly supported the challenging assignments of diastereotopic methyl carbons C29 and C30, the methyl carbons C26 and C27 attached to the olefin, and diastereotopic protons H2a and H2b. The presented computation approach can be potentially applied for the structure determinations of similar triterpenoid oximes and other large, complex molecules.

## Experimental section

Chemical reaction was carried out at International University, VNU HCM. ^1^H and ^13^C NMR spectra were recorded on a Bruker 400 MHz spectrometer at ambient temperature at University of Science, VNU HCM. ^1^H and ^13^C chemical shifts were reported in ppm using residual solvent peaks as an internal reference (CDCl_3_: 7.27 ppm for ^1^H NMR and 77.16 ppm for ^13^C NMR).

### Synthesis of 1

#### Procedure for the synthesis of oxime 1

2.1 g Hydroxylamine hydrochloride (30 mmol) and anhydrous sodium acetate (CH_3_COONa, 2.7 g, 33 mmol) were added to the solution of 4.4 g dipterocarpol (9.7 mmol) in 40 mL methanol at room temperature. The reaction progress was monitored by thin layer chromatography. Upon completion, the reaction mixture was placed in the refrigerator at 0–10 °C overnight for the product recrystallization. After filtered and washed with water, the crystallized oxime 1 was obtained as a white amorphous solid (3.95 g, 87% yield).

(8*R*,9*R*,10*R*,13*R*,14*R*,17*S*,*E*)-17-((*S*)-2-hydroxy-6-methylhept-5-en-2-yl)-4,4,8,10,14-pentamethylhexadecahydro-3*H*-cyclopenta[*a*]phenanthren-3-one oxime (1). *R*_f_ = 0.88 (20% EtOAc/Hexane); IR (KBr, *v*_max_, cm^−1^): 3307 (N–OH), 2938 (OH). ^1^H NMR (400 MHz, CDCl_3_) *δ* 5.12 (m, 1H), 2.98 (ddd, *J* = 15.4, 5.9, 3.9 Hz, 1H), 2.32–2.22 (m, 1H), 2.05 (m, 2H), 1.85–1.75 (m, 2H), 1.75–1.70 (m, 2H), 1.68 (s, 3H), 1.65 (d, *J* = 11.4 Hz, 3H), 1.62 (s, 3H), 1.56–1.42 (m, 8H), 1.38–1.19 (m, 6H), 1.14 (s, 6H), 1.12–1.05 (m, 2H), 1.05 (s, 3H), 0.98 (s, 3H), 0.94 (s, 3H), 0.86 (s, 3H). ^13^C NMR (101 MHz, CDCl_3_) *δ* 167.21, 131.68, 124.69, 75.46, 56.04, 50.29, 50.25, 49.77, 42.31, 40.48, 40.46, 40.38, 39.10, 37.18, 34.82, 31.14, 27.53, 27.29, 25.79, 25.42, 24.80, 22.86, 22.56, 21.80, 19.03, 17.75, 17.13, 16.34, 15.92, 15.40.

## Author contributions

Phong Q. Le & Nhu Q. Nguyen: performing the experimental section and experimental NMR analysis. Thien T. Nguyen: conceptualization, methodology, software, NMR analysis, investigation, writing – original draft, writing – review & editing, supervision.

## Conflicts of interest

There is no conflict to declare.

## Supplementary Material

RA-013-D3RA04688E-s001

RA-013-D3RA04688E-s002
