# Supplementary material for: DFT approach towards accurate prediction of 1H/13C NMR chemical shifts for dipterocarpol oxime
Source: RSC Adv. 2023 Oct 30;13(45):31811–9. doi: 10.1039/d3ra04688e (PMC10613955; doi:10.1039/d3ra04688e)
Supplement: RA-013-D3RA04688E-s001 [file RA-013-D3RA04688E-s001.pdf]

Electronic Supplementary Material (ESI) for RSC Advances.
This journal is © The Royal Society of Chemistry 2023

DFT approach towards accurate prediction of $^{1}H/^{13}C$ NMR chemical shifts for dipterocarpol oxime

Phong Q. Le[1], Trang T. Q. Phan[1], Thien T. Nguyen[2,3*]

[1] School of Biotechnology, International University, VNU-HCM, Quarter 6, Linh Trung Ward, Thu Duc City, Ho Chi Minh City, Vietnam

[2] Faculty of Pharmacy, College of Medicine and Pharmacy, Duy Tan University, Da Nang 550000, Vietnam

[3] Institute of Research and Development, Duy Tan University, Da Nang 550000, Vietnam

***Corresponding Author:** nguyentrongthien@duytan.edu.vn

Supporting Information

Table of content

1. Screening DFT methods for $^{1}H/^{13}C$ calculations
2. Boltzmann weighing calculations for major contributing conformers
3. $^{1}H/^{13}C$ NMR of dipterocarpol and $^{1}H/^{13}C$ NMR and COSY spectra of oxime
4. Cartesian coordinates of major contributing conformers for oxime 1 optimized at the level PCM($CHCl_3$)/B3LYP/6-31G(d,p)

1. Screening DFT methods for 1H/13C calculations of oxime **1**

Table S1. Screening density functional methods and basis sets for ^{13}C calculations

	B97D		BPV86		HCTH		LSDA		M062X		PBEPBE		TPSSTPSS	
	r^2	RMSE	r^2	RMSE	r^2	RMSE	r^2	RMSE	r^2	RMSE	r^2	RMSE	r^2	RMSE
6-31G	0.9958	2.32	0.9951	2.50	0.9969	2.00	0.9943	2.71	0.9984	1.41	0.9957	2.34	0.9968	2.01
6-31G(d,p)	0.9957	2.35	0.9951	2.51	0.9970	1.97	0.9942	2.74	0.9984	1.42	0.9955	2.40	0.9971	1.94
6-31+G(d,p)	0.9970	1.98	0.9965	2.11	0.9963	2.17	0.9959	2.29	0.9979	1.64	0.9968	2.03	0.9975	1.78
6-31++G(d,p)	0.9972	1.90	0.9972	1.90	0.9971	1.94	0.9968	2.03	0.9978	1.70	0.9973	1.86	0.9978	1.67
6-311G(d,p)	0.9958	2.31	0.9952	2.48	0.9962	2.21	0.9940	2.78	0.9978	1.68	0.9953	2.45	0.9968	2.03
6-31G(d,3p)	0.9948	2.58	0.9941	2.76	0.9957	2.35	0.9930	3.00	0.9983	1.46	0.9945	2.65	0.9964	2.16
6-31G(3d,p)	0.9873	4.06	0.9871	4.08	0.9898	3.64	0.9852	4.39	0.9949	2.55	0.9881	3.94	0.9903	3.55
6-31G(3d,3p)	0.9823	4.80	0.9823	4.80	0.9853	4.38	0.9797	5.15	0.9895	3.68	0.9833	4.66	0.9860	4.26
cc-pVDZ	0.9946	2.62	0.9940	2.77	0.9948	2.59	0.9932	2.96	0.9983	1.49	0.9943	2.70	0.9961	2.24
DGDZVP	0.9973	1.87	0.9968	2.02	0.9979	1.64	0.9958	2.31	0.9977	1.73	0.9971	1.93	0.9982	1.54
DGDZVP2	0.9949	2.55	0.9949	2.57	0.9956	2.37	0.9942	2.72	0.9980	1.62	0.9953	2.45	0.9964	2.15

All values are in ppm.

Table S2. Screening density functional methods and basis sets for ^{1}H calculations

	B97D		B3PW91		CAMB3LYP		M062X		mPW1PW91		TPSSTPSS		*ω*B97XD	
	r^2	RMSE	r^2	RMSE	r^2	RMSE	r^2	RMSE	r^2	RMSE	r^2	RMSE	r^2	RMSE
6-31G	0.9885	0.126	0.9913	0.109	0.9907	0.113	0.9865	0.137	0.9911	0.111	0.9913	0.109	0.9853	0.143
6-31G(d,p)	0.9850	0.144	0.9877	0.130	0.9869	0.135	0.9805	0.165	0.9873	0.133	0.9878	0.130	0.9845	0.159
6-31+G(d,p)	0.9864	0.137	0.9887	0.125	0.9887	0.125	0.9804	0.165	0.9886	0.126	0.9893	0.122	0.9776	0.177
6-31++G(d,p)	0.9823	0.157	0.9844	0.147	0.9862	0.139	0.9831	0.153	0.9843	0.148	0.9836	0.151	0.9864	0.137
6-311G(d,p)	0.9828	0.155	0.9870	0.134	0.9861	0.139	0.9804	0.165	0.9866	0.136	0.9878	0.130	0.9868	0.135
6-31G(d,3p)	0.9836	0.151	0.9867	0.136	0.9856	0.141	0.9818	0.159	0.9864	0.137	0.9873	0.133	0.9835	0.151
6-31G(3d,p)	0.9669	0.216	0.9800	0.167	0.9804	0.165	0.9694	0.208	0.9802	0.166	0.9787	0.173	0.9835	0.162
6-31G(3d,3p)	0.9579	0.245	0.9789	0.172	0.9803	0.166	0.9462	0.279	0.9790	0.171	0.9766	0.181	0.9772	0.179
cc-pVDZ	0.9832	0.153	0.9858	0.140	0.9845	0.147	0.9790	0.171	0.9854	0.142	0.9868	0.135	0.9833	0.152
DGDZVP	0.9845	0.147	0.9853	0.143	0.9858	0.140	0.9820	0.158	0.9846	0.146	0.9865	0.137	0.9800	0.167
DGDZVP2	0.9867	0.136	0.9882	0.128	0.9876	0.131	0.9809	0.163	0.9879	0.130	0.9890	0.123	0.9830	0.154

All values are in ppm.

Table S3. NMR calculation times for the global minimum

	B3LYP	B3PW91	B97D	BPV86	CAM-B3LYP	HCTH	HSEH1PBE	LSDA	M062X	mPW1PW91	PBEPBE	TPSSTPSS	ωB97XD
6-31G	0.57	0.57	0.40	0.41	0.71	0.42	0.63	0.38	0.76	0.58	0.41	0.58	0.71
6-31G(d,p)	1.94	1.87	1.03	1.02	2.25	0.99	1.87	0.95	2.40	1.88	0.99	1.41	2.27
6-31G(d,3p)	4.53	4.53	2.40	2.15	5.57	2.32	4.47	1.91	5.46	4.55	2.16	2.89	5.61
6-31G(3d,p)	9.03	8.66	3.69	3.55	10.65	3.53	9.19	3.18	9.94	8.54	3.54	4.19	11.59
6-31G(3d,3p)	16.63	16.65	6.65	6.54	22.36	6.61	17.20	5.87	20.29	17.20	6.45	7.12	21.97
6-31+G(d,p)	7.36	6.92	2.70	2.73	8.84	2.97	6.81	2.40	8.65	6.87	2.72	3.71	9.01
6-31++G(d,p)	11.18	12.56	4.51	4.46	14.84	4.41	11.20	4.68	13.13	11.28	4.45	5.32	15.21
6-311G(d,p)	4.02	4.14	1.88	1.87	4.69	1.90	4.15	1.69	4.90	4.07	1.87	2.50	4.89
cc-pVDZ	3.52	3.50	1.85	1.83	4.81	1.78	4.39	1.73	4.69	3.56	1.86	2.31	4.83
DGDZVP	2.59	2.39	1.36	1.33	3.27	1.32	2.81	1.32	3.00	2.41	2.92	1.72	3.25
DGDZVP2	4.92	4.60	2.31	2.27	6.15	2.27	5.09	2.10	5.16	4.33	2.27	2.79	6.03

Time values are in hour.

2. Boltzmann weighting calculations for major contributing conformers

Table S4. Boltzmann weighing of major contributing conformers

Conformer	Energy (Hartree)	Relative energy (kJ mol-1)	Boltzmann weighting	Percentage contribution (%)
1	-1379.10108537	0.00	1.00	37.17
2	-1379.10021687	2.28	0.40	14.81
3	-1379.10008163	2.64	0.35	12.83
4	-1379.09979053	3.40	0.25	9.42
5	-1379.09916033	5.05	0.13	4.83
6	-1379.09904181	5.37	0.11	4.26
7	-1379.09899411	5.49	0.11	4.05
8	-1379.09887102	5.81	0.10	3.56
9	-1379.09863435	6.44	0.07	2.77
10	-1379.09811452	7.80	0.04	1.60
11	-1379.09782408	8.56	0.03	1.17
12	-1379.09776823	8.71	0.03	1.11
13	-1379.09762826	9.08	0.03	0.95
14	-1379.09746482	9.51	0.02	0.80
15	-1379.09730329	9.93	0.02	0.68

3. $^{1}H/^{13}C$ NMR spectra of dipterocarpol and $^{1}H/^{13}C$ NMR and COSY spectra of oxime **1**

a. $^{1}H/^{13}C$ NMR spectra of dipterocarpol

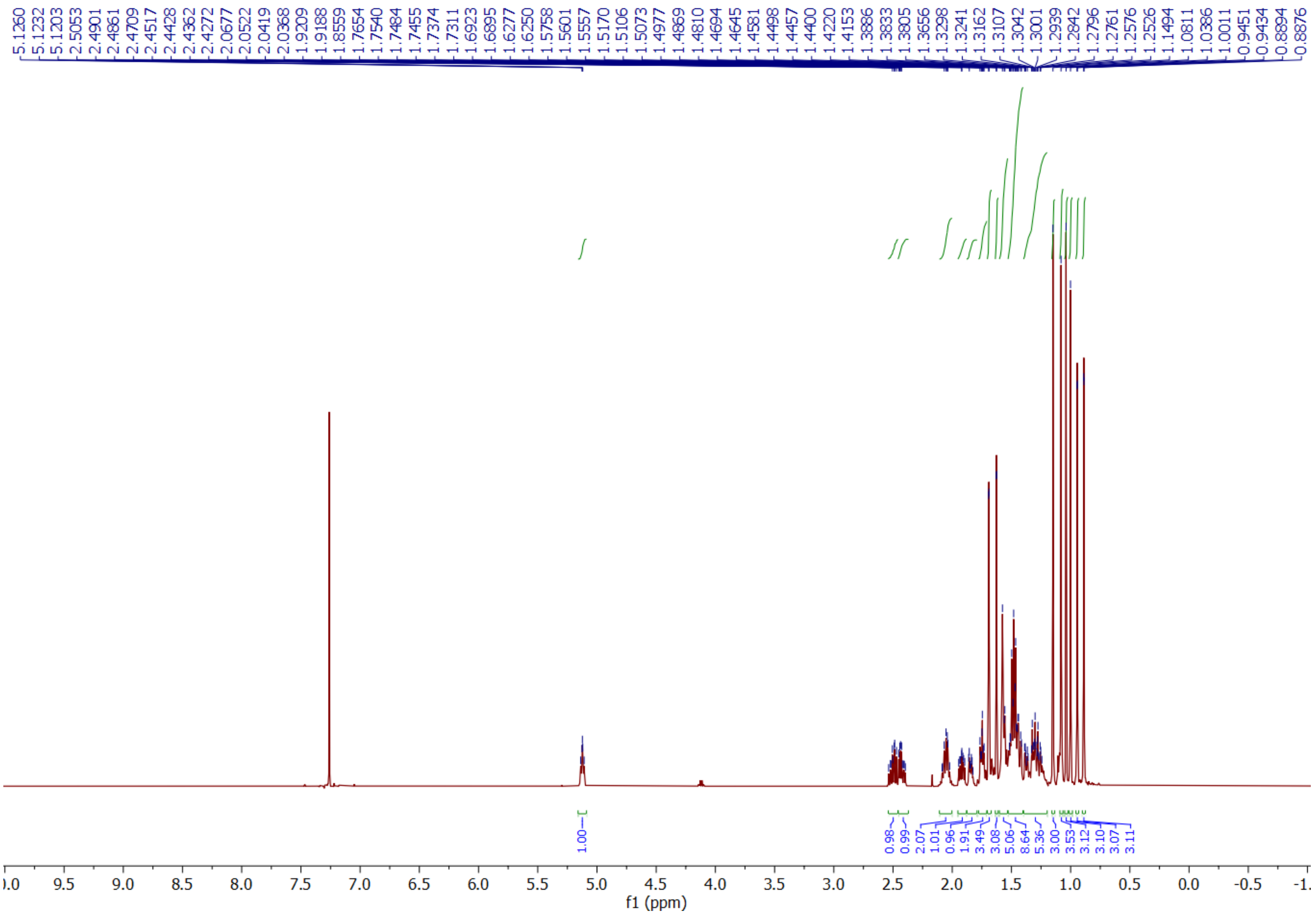

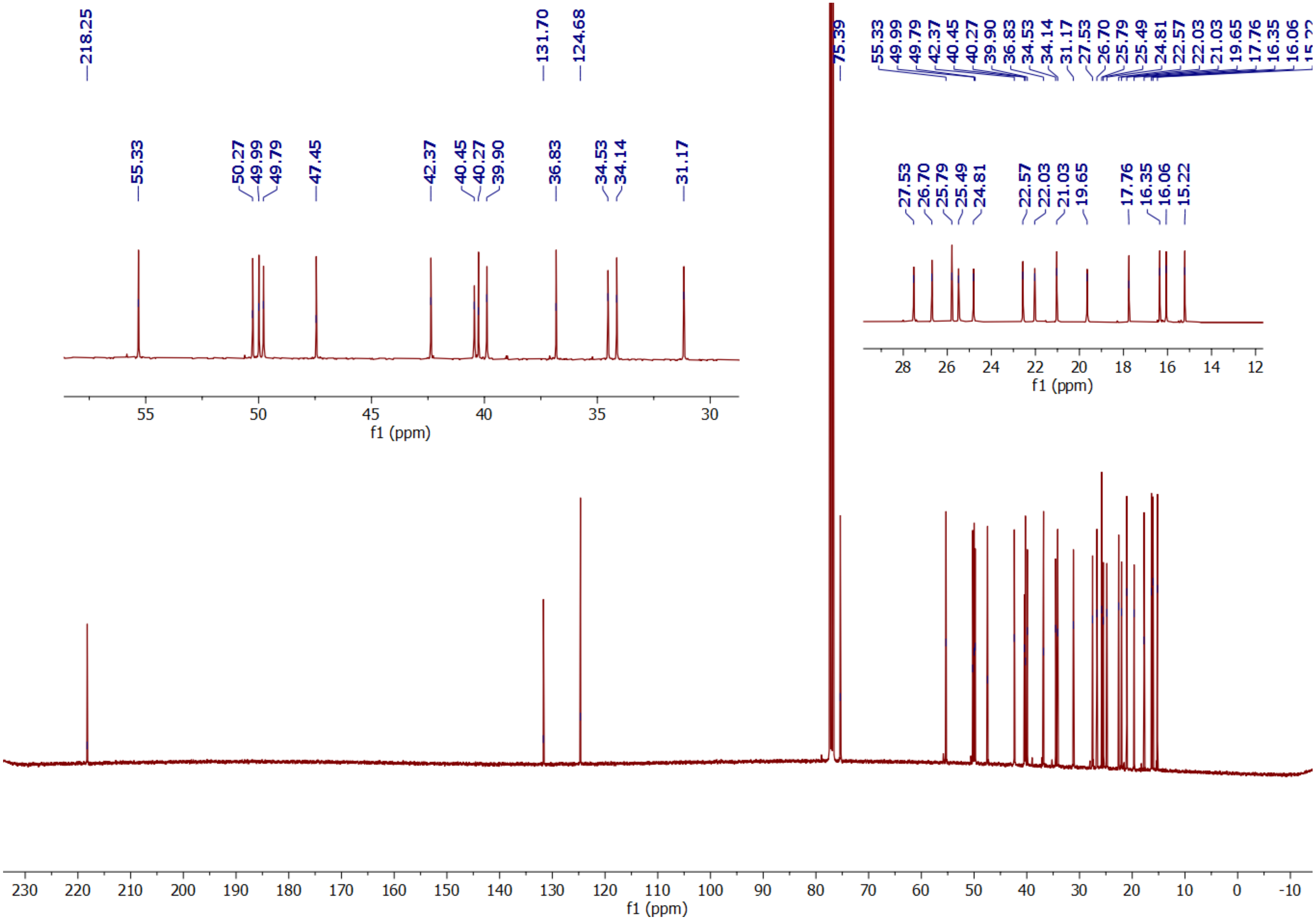

218.25
131.70
124.68
75.39
55.33
49.99
49.79
42.37
40.45
40.27
39.90
36.83
34.53
34.14
31.17
27.53
26.70
25.79
25.49
24.81
22.57
22.03
21.03
19.65
17.76
16.35
16.06
55.33
50.27
49.99
49.79
47.45
42.37
40.45
40.27
39.90
36.83
34.53
34.14
31.17
f1 (ppm)
27.53
26.70
25.79
25.49
24.81
22.57
22.03
21.03
19.65
17.76
16.35
16.06
15.22
f1 (ppm)
f1 (ppm)

b. $^{1}H/^{13}C$ NMR and COSY spectra of oxime **1**

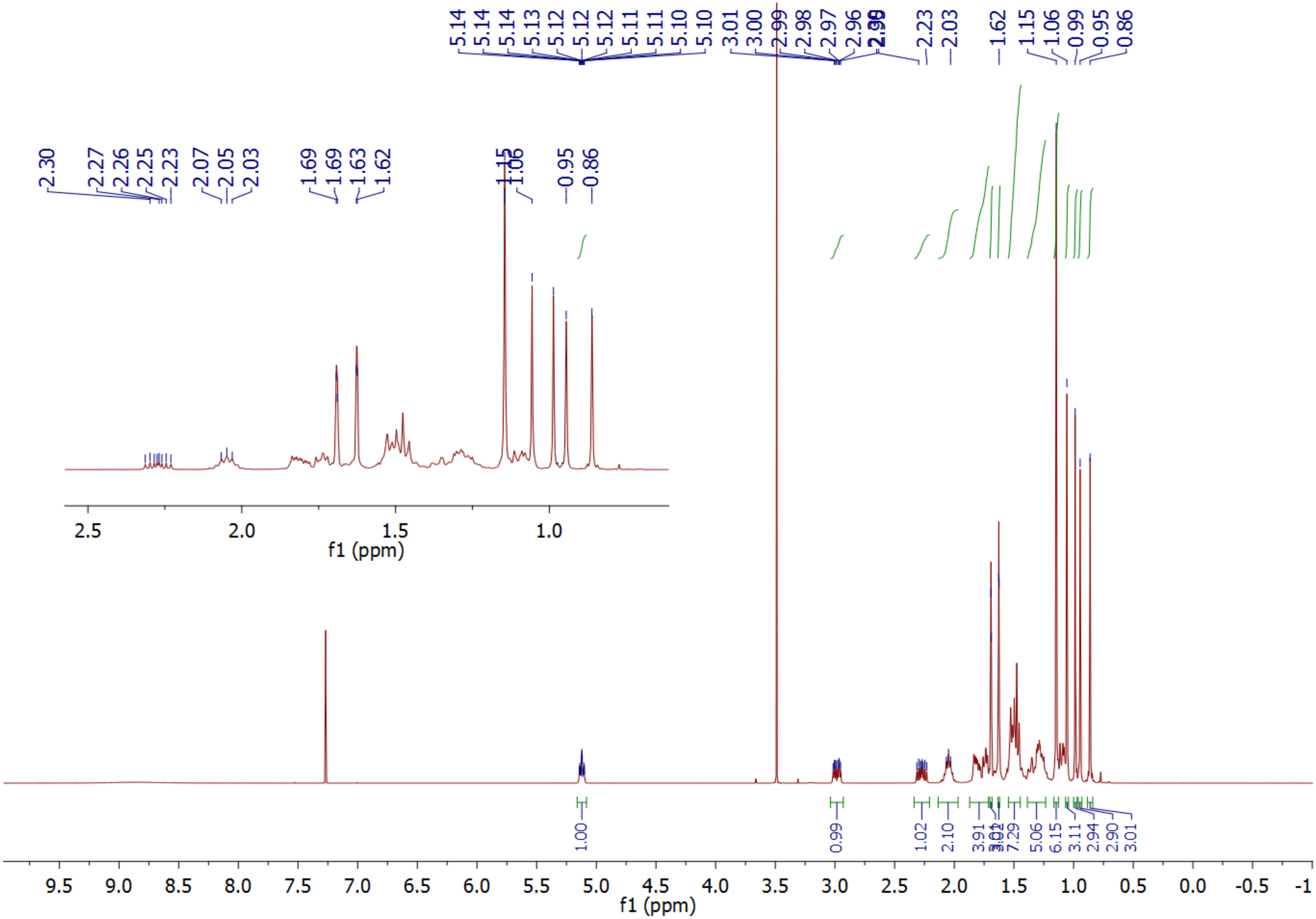

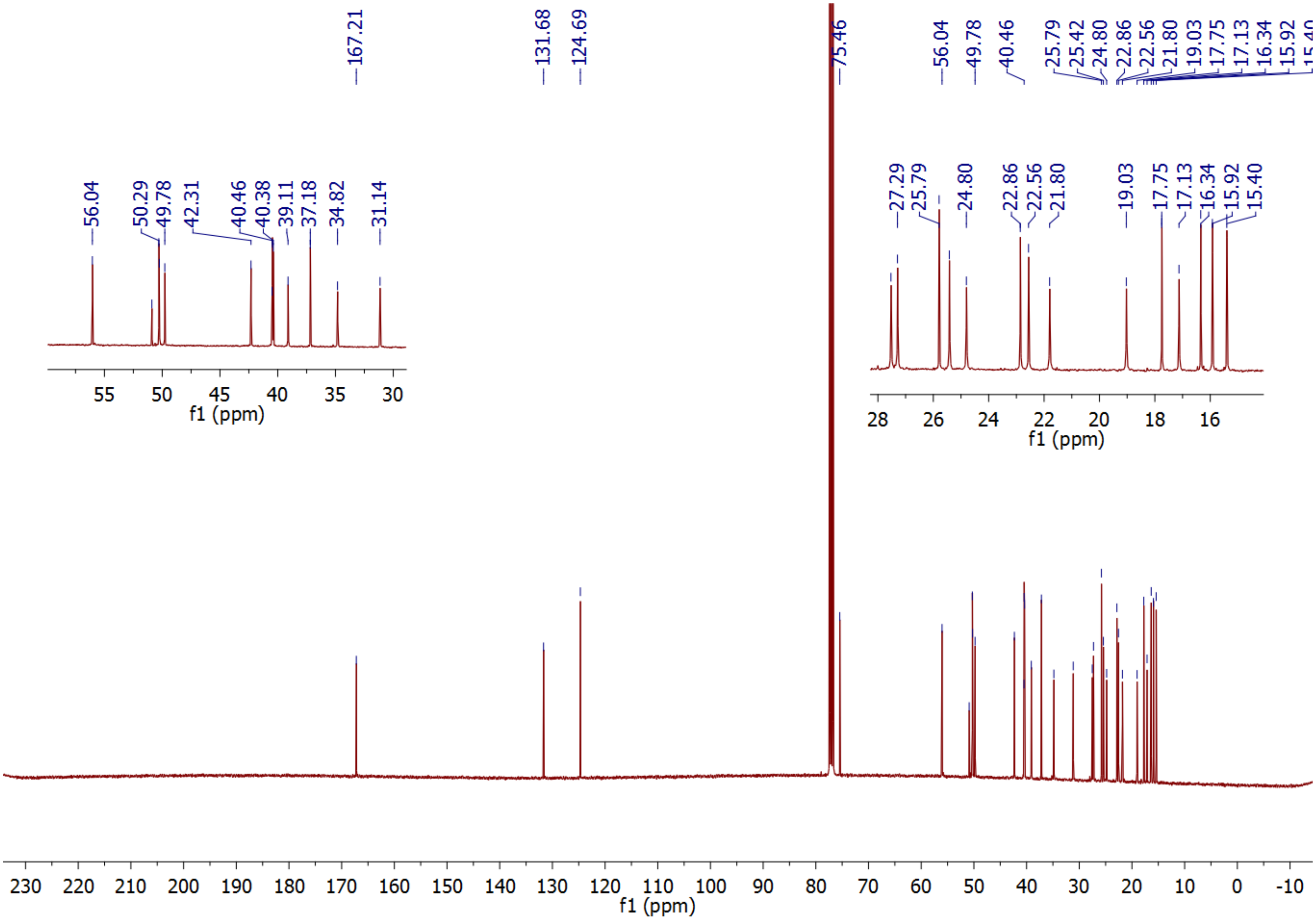
167.21
131.68
124.69
75.46
56.04
49.78
40.46
25.79
25.42
24.80
22.86
22.56
21.80
19.03
17.75
17.13
16.34
15.92
56.04
50.29
49.78
42.31
40.46
40.38
39.11
37.18
34.82
31.14
f1 (ppm)
27.29
25.79
24.80
22.86
22.56
21.80
19.03
17.75
17.13
16.34
15.92
15.40
f1 (ppm)
f1 (ppm)

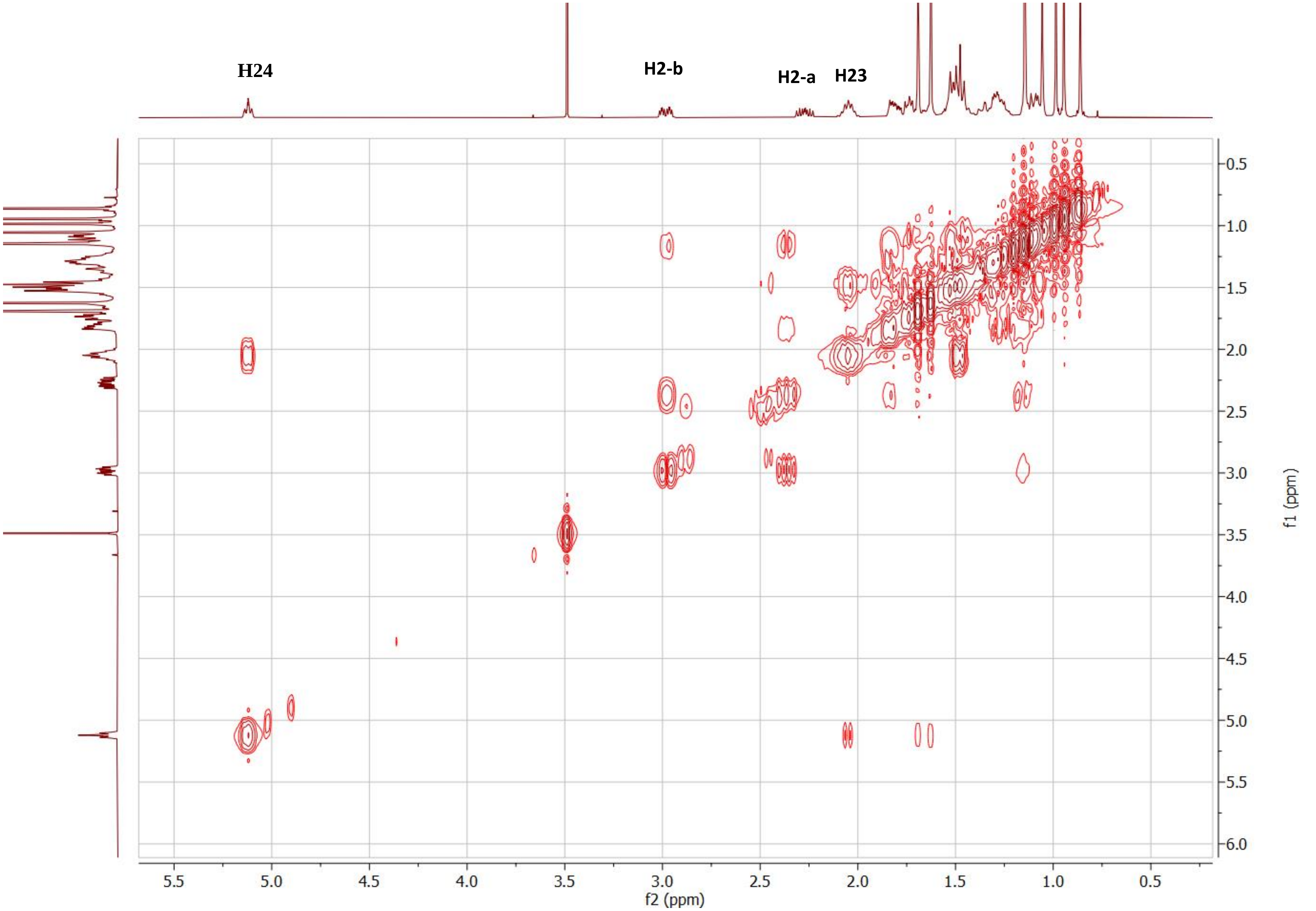
H24
H2-b
H2-a
H23
f2 (ppm)
f1 (ppm)

4. Cartesian coordinates of conformers for oxime 1 optimized at the level PCM($CHCl_3$)/B3LYP/6-31G(d,p

Conformer 1: E = -1379.10108537 Hartree

C	-2.6764	-3.382	0.5594
C	-2.4374	-2.5823	1.8736
C	-1.0508	-2.9617	2.5465
C	-2.4205	-1.0063	1.5772
C	-3.588	-2.9616	2.8401
C	-0.9418	-4.4381	3.14
C	0.157	-2.5088	1.6799
C	-2.3877	-0.1633	2.8964
C	-1.2246	-0.678	0.6389
C	-3.5738	-0.4285	0.7277
C	-3.5081	-4.3896	3.3925
C	-0.8462	-5.5028	2.0225
C	-2.1661	-4.641	4.1146
C	0.3531	-4.5335	4.0045
C	0.1107	-1.0241	1.2811
C	-1.466	0.7825	0.1988
C	-3.0113	0.8631	0.0992
C	-2.142	-5.933	5.0083
C	0.4674	-5.7987	4.8486
C	-0.6955	1.2897	-1.0602
C	-3.2611	-5.843	6.0816
C	-2.3661	-7.2506	4.2506
C	-0.7647	-5.9999	5.7132
C	-0.8602	0.3485	-2.2829
C	-1.12	2.7349	-1.3698
O	0.6887	1.3324	-0.6861
N	-0.719	-6.2852	6.9839
C	-0.3558	0.8558	-3.6472
C	1.1417	0.9599	-3.746
C	1.8852	2.0206	-4.1181
C	3.3905	1.9251	-4.1459
C	1.3509	3.3611	-4.5431
O	0.5864	-6.3593	7.4741
H	-1.8596	-3.2868	-0.1589
H	-2.8121	-4.4483	0.7294
H	-3.5994	-3.0769	0.0598
H	-0.9797	-2.338	3.4467
H	-3.5933	-2.3018	3.7127
H	-4.5576	-2.8195	2.3465
H	1.0963	-2.6668	2.2188
H	0.2223	-3.107	0.7658
H	-3.3141	-0.2822	3.4667
H	-1.554	-0.4151	3.5537
H	-2.3094	0.9124	2.7137
H	-1.3113	-1.2932	-0.2623
H	-4.4788	-0.2312	1.3119
H	-3.8623	-1.0884	-0.0936
H	-4.3461	-4.4963	4.0877
H	-3.6841	-5.1256	2.604
H	-0.3917	-5.1092	1.1105
H	-0.2197	-6.3536	2.3007
H	-1.8164	-5.923	1.7529
H	-2.0801	-3.8176	4.8446
H	1.2421	-4.4816	3.3651
H	0.4024	-3.6694	4.6808
H	0.2974	-0.397	2.1599
H	0.9319	-0.8324	0.5826
H	-1.1429	1.4295	1.0267
H	-3.3754	0.9586	-0.9281
H	-3.3711	1.7422	0.6466
H	1.3743	-5.7145	5.4537
H	0.5977	-6.6916	4.2309
H	-3.1964	-6.6645	6.8054
H	-4.2604	-5.9092	5.6406
H	-3.1955	-4.9061	6.6464
H	-3.2809	-7.2222	3.6505
H	-1.5348	-7.5156	3.5961
H	-2.4635	-8.0875	4.9535
H	-1.9218	0.1069	-2.4118
H	-0.3431	-0.5981	-2.0794
H	-0.4371	3.2128	-2.0791
H	-1.0794	3.3522	-0.4645
H	-2.1338	2.7881	-1.7774
H	1.185	1.7493	-1.4123
H	-0.8646	1.7866	-3.9065
H	-0.6715	0.1283	-4.4067
H	1.6622	0.0369	-3.4839
H	3.8288	2.6716	-3.4753
H	3.7515	0.9413	-3.8282
H	3.7634	2.1048	-5.1596
H	1.6835	4.1356	-3.8441
H	1.7241	3.6147	-5.5412
H	0.2612	3.4035	-4.5888
H	0.4468	-6.5725	8.4162

Conformer 2: E = -1379.10021687 Hartree

C	-1.9976	-3.4932	0.4915
C	-2.0803	-2.6831	1.8176
C	-0.8474	-2.9827	2.7704
C	-2.092	-1.1113	1.5096
C	-3.3821	-3.1249	2.5326
C	-0.7739	-4.4547	3.376
C	0.4835	-2.4361	2.1862
C	-2.3784	-0.2669	2.799
C	-0.7503	-0.7092	0.8273
C	-3.079	-0.6077	0.442
C	-3.3392	-4.5485	3.1023
C	-0.3892	-5.4993	2.3032
C	-2.1581	-4.7356	4.0804
C	0.32	-4.4848	4.4873
C	0.4137	-0.951	1.786
C	-0.981	0.727	0.2859
C	-2.5175	0.7559	0.0126
C	-2.2428	-6.0347	4.9601
C	0.3324	-5.7506	5.3382
C	-0.1565	1.1531	-0.9792
C	-3.5607	-6.0209	5.7815
C	-2.2306	-7.3538	4.1726
C	-1.0367	-6.0326	5.9318

C	-0.4804	2.6419	-1.3099
C	1.3481	0.9448	-0.7819
O	-0.5655	0.3113	-2.0627
N	-1.2349	-6.3324	7.1844
C	0.3036	3.3279	-2.4441
C	0.016	2.7747	-3.8126
C	0.8886	2.3101	-4.7286
C	0.3954	1.7834	-6.0535
C	2.3839	2.2624	-4.5713
O	-0.055	-6.3396	7.9311
H	-1.0496	-3.3619	-0.0343
H	-2.1227	-4.5643	0.6378
H	-2.8037	-3.2283	-0.1977
H	-1.0093	-2.3703	3.666
H	-3.6002	-2.4633	3.377
H	-4.2364	-3.0366	1.8497
H	1.2906	-2.5328	2.919
H	0.7898	-3.0165	1.3103
H	-3.4241	-0.367	3.1093
H	-1.7558	-0.5393	3.652
H	-2.2153	0.8047	2.6509
H	-0.5801	-1.3389	-0.054
H	-4.1075	-0.525	0.8083
H	-3.1008	-1.2457	-0.4451
H	-4.2929	-4.7009	3.6164
H	-3.315	-5.2903	2.2999
H	0.215	-5.0676	1.5018
H	0.2198	-6.3135	2.7035
H	-1.2574	-5.9749	1.8444
H	-2.2687	-3.9174	4.813
H	1.3162	-4.3749	4.0431
H	0.1794	-3.6267	5.1584
H	0.3203	-0.3284	2.6826
H	1.3701	-0.678	1.3356
H	-0.751	1.4414	1.0879
H	-2.7793	0.9425	-1.0336
H	-2.9773	1.5615	0.5974
H	1.0894	-5.6208	6.1166
H	0.6389	-6.6276	4.7614
H	-3.5981	-6.8459	6.5032
H	-4.4431	-6.1382	5.145
H	-3.6662	-5.0886	6.3479
H	-3.0028	-7.37	3.3973
H	-1.2687	-7.5639	3.7031
H	-2.4219	-8.2031	4.8406
H	-0.3153	3.2395	-0.4031
H	-1.5455	2.738	-1.5543
H	1.9218	1.2812	-1.6504
H	1.5903	-0.1192	-0.7014
H	1.718	1.4747	0.101
H	-0.0049	0.5161	-2.8315
H	1.3687	3.3398	-2.2052
H	0.0009	4.3835	-2.461
H	-1.0456	2.7677	-4.0666
H	0.697	0.7385	-6.1804
H	-0.6954	1.8266	-6.139
H	0.817	2.3707	-6.8758
H	2.8653	2.8027	-5.3935
H	2.7409	2.7101	-3.6423
H	2.7297	1.2237	-4.5926
H	-0.371	-6.576	8.8238

Conformer 3: E = -1379.10008163 Hartree

C	-1.8495	-3.2374	0.3021
C	-2.0949	-2.5411	1.6728
C	-0.891	-2.7852	2.6781
C	-2.254	-0.9573	1.4777
C	-3.3822	-3.168	2.2656
C	-0.7058	-4.2819	3.1975
C	0.413	-2.0868	2.2016
C	-2.7044	-0.2476	2.7988
C	-0.9223	-0.3773	0.9221
C	-3.2207	-0.4677	0.3769
C	-3.2219	-4.617	2.7405
C	-0.1511	-5.2107	2.0924
C	-2.0936	-4.7465	3.7873
C	0.3152	-4.2777	4.3767
C	0.2351	-0.592	1.8858
C	-1.2701	1.0696	0.5126
C	-2.7265	0.9433	-0.0035
C	-2.0974	-6.1023	4.5812
C	0.4064	-5.59	5.1488
C	-0.2922	1.7986	-0.4584
C	-3.4574	-6.2727	5.3117
C	-1.902	-7.3593	3.7198
C	-0.96	-6.0446	5.6307
C	0.0084	0.9937	-1.7452
C	-0.8279	3.2055	-0.7759
O	0.9584	1.9806	0.2226
N	-1.2026	-6.4439	6.8472
C	0.9181	1.7141	-2.751
C	1.261	0.815	-3.9107
C	0.7357	0.8323	-5.1516
C	1.2262	-0.1261	-6.2062
C	-0.3603	1.753	-5.6142
O	-0.0763	-6.3829	7.6704
H	-0.8992	-2.961	-0.1589
H	-1.8524	-4.3235	0.3704
H	-2.6459	-3.018	-0.4138
H	-1.1598	-2.2467	3.5959
H	-3.7216	-2.6019	3.138
H	-4.1984	-3.1163	1.5341
H	1.1905	-2.1622	2.9681
H	0.8114	-2.5801	1.3096
H	-2.0429	-0.4414	3.6446
H	-2.7553	0.8409	2.7028
H	-3.7163	-0.5492	3.0865
H	-0.6706	-0.9108	0.0004
H	-4.2668	-0.4542	0.7007
H	-3.1746	-1.0806	-0.5262
H	-4.1845	-4.9057	3.1731
H	-3.0649	-5.2915	1.895
H	0.4578	-4.672	1.3629
H	0.5084	-5.9887	2.4846
H	-0.9362	-5.7344	1.5448
H	-2.3351	-3.9931	4.5569
H	1.3203	-4.0405	4.0089
H	0.0458	-3.4845	5.0871
H	0.0813	-0.0315	2.8145
H	1.1688	-0.2209	1.4505
H	-1.2853	1.6722	1.4315
H	-2.8166	1.084	-1.0847
H	-3.3535	1.7081	0.4696
H	1.0972	-5.4381	5.9827
H	0.836	-6.392	4.5422
H	-3.4537	-7.1431	5.9789

H	-4.283	-6.4341	4.6118
H	-3.6922	-5.3946	5.9241
H	-2.6204	-7.401	2.8952
H	-0.8971	-7.4419	3.3036
H	-2.0447	-8.2654	4.3221
H	-0.9265	0.72	-2.2479
H	0.5145	0.0593	-1.4719
H	-0.0544	3.8362	-1.2277
H	-1.1279	3.7229	0.143
H	-1.6905	3.1743	-1.4484
H	0.7874	2.4967	1.0298
H	1.8561	2.0121	-2.2672
H	0.4525	2.6383	-3.1032
H	2.041	0.0868	-3.6863
H	2.027	-0.7758	-5.8377
H	0.407	-0.7682	-6.5461
H	1.6165	0.4265	-7.0671
H	-0.7993	2.3437	-4.8069
H	0.024	2.4456	-6.37
H	-1.1749	1.1727	-6.0609
H	-0.4205	-6.7072	8.5241

Conformer 4: E = -1379.09979053 Hartree

C	-2.5928	-3.4247	0.7283
C	-2.4953	-2.4937	1.9716
C	-1.1445	-2.7158	2.7686
C	-2.5439	-0.954	1.5218
C	-3.6839	-2.851	2.9004
C	-0.9786	-4.0944	3.5398
C	0.0939	-2.2904	1.9341
C	-2.6634	0.0107	2.7492
C	-1.2983	-0.6394	0.6432
C	-3.6573	-0.5334	0.5371
C	-3.5673	-4.2315	3.5525
C	-0.6348	-5.2674	2.593
C	-2.2779	-4.3638	4.3866
C	0.2351	-3.9534	4.5306
C	0.0046	-0.8503	1.4021
C	-1.5851	0.7574	0.0518
C	-3.1188	0.7247	-0.1763
C	-2.2328	-5.6709	5.2654
C	0.0772	-4.6632	5.8734
C	-0.7419	1.2017	-1.1839
C	-3.0846	-5.4688	6.5394
C	-2.7758	-6.9255	4.5503
C	-0.7837	-5.9014	5.7393
C	-0.7399	0.1444	-2.3197
C	-1.2277	2.5792	-1.6644
O	0.5991	1.3759	-0.7046
N	-0.3953	-7.1092	6.0313
C	-0.1496	0.56	-3.6807
C	1.3413	0.7601	-3.6661
C	2.0469	1.8326	-4.0761
C	3.5517	1.8416	-3.971
C	1.4693	3.0882	-4.6702
O	0.9344	-7.1519	6.456
H	-1.7461	-3.3283	0.0461
H	-2.6539	-4.4788	0.9869
H	-3.5066	-3.2405	0.1568
H	-1.1805	-1.9928	3.5933
H	-3.7669	-2.1318	3.7207
H	-4.6275	-2.7926	2.344
H	1.0078	-2.3683	2.5326
H	0.2293	-2.9638	1.0813
H	-1.8592	-0.1084	3.477
H	-2.6541	1.067	2.4646
H	-3.6152	-0.1276	3.2713
H	-1.2858	-1.3358	-0.2014
H	-4.6146	-0.3374	1.0316
H	-3.8438	-1.2866	-0.232
H	-4.4408	-4.3645	4.2019
H	-3.6335	-5.0166	2.7947
H	0.1113	-4.9876	1.8445
H	-0.2	-6.1124	3.1376
H	-1.5026	-5.6634	2.0682
H	-2.3223	-3.5255	5.1016
H	1.1529	-4.3118	4.0454
H	0.4275	-2.8977	4.7616
H	0.0958	-0.1399	2.231
H	0.8622	-0.6749	0.7444
H	-1.3717	1.4945	0.8388
H	-3.3984	0.6928	-1.2337
H	-3.5746	1.6281	0.2459
H	-0.4223	-3.9958	6.5861
H	1.0629	-4.8721	6.296
H	-3.0154	-6.3386	7.2035
H	-4.142	-5.319	6.2985
H	-2.7458	-4.6018	7.1176
H	-3.8217	-6.804	4.2506
H	-2.1949	-7.1837	3.6623
H	-2.7462	-7.8029	5.2079
H	-1.7691	-0.1811	-2.5111
H	-0.1853	-0.7415	-1.9843
H	-0.5171	3.0378	-2.3591
H	-1.3049	3.2773	-0.8225
H	-2.2036	2.5232	-2.1558
H	1.1296	1.7596	-1.4247
H	-0.6908	1.4252	-4.0694
H	-0.351	-0.2539	-4.3898
H	1.8933	-0.0964	-3.2746
H	3.8815	2.6744	-3.3412
H	3.9443	0.9183	-3.5321
H	4.0006	1.9552	-4.9632
H	1.9134	3.2771	-5.6535
H	0.3876	3.0487	-4.8102
H	1.6886	3.944	-4.0233
H	1.0703	-8.1026	6.6299

Conformer 5: E = -1379.09916033 Hartree

C	-3.3668	-3.2752	0.7819
C	-2.4634	-2.4616	1.7539
C	-1.1047	-3.2185	2.0667
C	-2.0954	-1.0376	1.1158
C	-3.2615	-2.298	3.0724
C	-1.2309	-4.562	2.9159
C	-0.1938	-3.3118	0.8128
C	-1.3646	-0.1091	2.1443
C	-1.2576	-1.2532	-0.1794
C	-3.2556	-0.2056	0.5314
C	-3.4152	-3.5895	3.8848
C	-1.8438	-5.7131	2.0851
C	-2.0425	-4.2118	4.2232
C	0.1954	-5.0038	3.3668
C	0.0566	-1.956	0.1301
C	-1.2204	0.1335	-0.86
C	-2.59	0.7621	-0.462

C	-2.0886	-5.3206	5.3355
C	0.2209	-6.1247	4.4011
C	-0.9413	0.1969	-2.3917
C	-2.6871	-4.7244	6.6387
C	-2.9361	-6.5539	4.988
C	-0.636	-5.7838	5.6076
C	0.5065	-0.1678	-2.8155
C	-1.9007	-0.6849	-3.2119
O	-1.2032	1.5407	-2.8334
N	-0.2377	-5.9213	6.8407
C	1.6723	0.4867	-2.0532
C	1.7815	1.9738	-2.2481
C	1.81	2.9413	-1.31
C	1.9171	4.3911	-1.713
C	1.752	2.7246	0.1778
O	1.0741	-6.3905	6.9353
H	-2.8618	-3.5535	-0.1453
H	-3.7379	-4.2	1.219
H	-4.2756	-2.7278	0.518
H	-0.5497	-2.5606	2.7473
H	-2.7684	-1.5803	3.7351
H	-4.2558	-1.884	2.8629
H	0.7851	-3.7217	1.0799
H	-0.6226	-3.9952	0.0732
H	-2.0419	0.2015	2.9463
H	-0.4907	-0.5709	2.6061
H	-1.0037	0.8225	1.6991
H	-1.8151	-1.9115	-0.853
H	-3.8284	0.3299	1.2956
H	-3.9644	-0.814	-0.0357
H	-3.9498	-3.3145	4.7988
H	-4.0624	-4.3027	3.3681
H	-1.6431	-5.6071	1.0165
H	-1.4345	-6.6911	2.3494
H	-2.9242	-5.7988	2.2115
H	-1.4712	-3.3936	4.6947
H	0.7809	-5.3383	2.5025
H	0.7261	-4.1402	3.7899
H	0.6947	-1.333	0.7664
H	0.6214	-2.1426	-0.7863
H	-0.4585	0.75	-0.3713
H	-3.2564	0.9445	-1.31
H	-2.4202	1.7361	0.0132
H	1.2637	-6.2939	4.683
H	-0.1379	-7.071	3.9869
H	-2.6188	-5.4292	7.4762
H	-3.7499	-4.4865	6.532
H	-2.1585	-3.812	6.9376
H	-3.9513	-6.2763	4.6879
H	-2.4997	-7.165	4.1967
H	-3.0234	-7.2187	5.8566
H	0.6284	0.0829	-3.8793
H	0.6382	-1.2541	-2.7519
H	-1.776	-0.4928	-4.2847
H	-2.9494	-0.4647	-2.9902
H	-1.7282	-1.7521	-3.0431
H	-0.6824	2.1434	-2.2735
H	2.6037	0.0482	-2.4352
H	1.6333	0.2056	-1.0002
H	1.8508	2.2728	-3.2956
H	1.9529	4.5199	-2.7999
H	1.053	4.9513	-1.341
H	2.8268	4.8347	-1.2954
H	2.6185	3.1904	0.6592
H	0.8442	3.1801	0.5872
H	1.7525	1.6726	0.4683
H	1.2133	-6.4362	7.9002

Conformer 6: E = -1379.09904181 Hartree

C	-1.8217	-3.2381	0.3136
C	-2.1018	-2.5569	1.6854
C	-0.9105	-2.7884	2.7085
C	-2.2851	-0.9745	1.4998
C	-3.3878	-3.2105	2.2519
C	-0.7083	-4.2856	3.2198
C	0.3889	-2.0645	2.2582
C	-2.7686	-0.283	2.8188
C	-0.955	-0.3671	0.9702
C	-3.2428	-0.4934	0.3876
C	-3.2104	-4.6601	2.7188
C	-0.12	-5.1966	2.1173
C	-2.0974	-4.778	3.7833
C	0.293	-4.2731	4.4156
C	0.1908	-0.5706	1.9506
C	-1.3216	1.0776	0.5666
C	-2.7662	0.9278	0.0235
C	-2.0914	-6.1394	4.5675
C	0.394	-5.5896	5.1793
C	-0.3388	1.8332	-0.3793
C	-3.4599	-6.3372	5.2748
C	-1.8615	-7.3867	3.7004
C	-0.9719	-6.0711	5.6355
C	0.0191	1.025	-1.6513
C	-0.9103	3.2231	-0.7097
O	0.8672	2.0454	0.3711
N	-1.2269	-6.485	6.8446
C	0.8636	1.7785	-2.6887
C	1.2525	0.8749	-3.8291
C	0.7358	0.8458	-5.0734
C	1.2754	-0.1113	-6.1055
C	-0.395	1.7082	-5.5638
O	-0.115	-6.4121	7.6861
H	-0.8689	-2.9416	-0.1294
H	-1.8068	-4.3246	0.3737
H	-2.6098	-3.0269	-0.4138
H	-1.2033	-2.2613	3.6255
H	-3.7515	-2.657	3.1225
H	-4.1924	-3.1673	1.5071
H	1.1551	-2.1326	3.0366
H	0.8099	-2.5442	1.3692
H	-2.1168	-0.4709	3.6734
H	-2.8379	0.8051	2.7296
H	-3.7792	-0.6048	3.0887
H	-0.6793	-0.8879	0.0478
H	-4.2937	-0.4992	0.6955
H	-3.1732	-1.0995	-0.5185
H	-4.1748	-4.9685	3.1332
H	-3.0278	-5.3254	1.8712
H	0.4914	-4.6423	1.4017
H	0.546	-5.9664	2.5145
H	-0.8871	-5.7294	1.5531
H	-2.3644	-4.0345	4.5541
H	1.2997	-4.016	4.0661
H	-0.0014	-3.4902	5.1274
H	0.0132	-0.0199	2.8809
H	1.1249	-0.1811	1.5327

H	-1.3627	1.6728	1.4897
H	-2.8381	1.0711	-1.0589
H	-3.4154	1.6794	0.4879
H	1.0685	-5.4325	6.0255
H	0.8471	-6.3796	4.5739
H	-3.4528	-7.2127	5.9354
H	-4.2716	-6.5065	4.5607
H	-3.7185	-5.4679	5.8901
H	-2.5665	-7.4346	2.8647
H	-0.8492	-7.4492	3.2991
H	-1.9978	-8.2993	4.2943
H	-0.8966	0.6725	-2.1407
H	0.5912	0.136	-1.3562
H	-0.1539	3.8737	-1.1622
H	-1.2295	3.737	0.2048
H	-1.7661	3.1648	-1.3889
H	1.4357	2.6609	-0.1219
H	1.787	2.1499	-2.2292
H	0.3309	2.66	-3.0544
H	2.0616	0.1844	-3.5884
H	2.1017	-0.7174	-5.7193
H	0.4877	-0.7957	-6.4371
H	1.6468	0.4407	-6.9752
H	-0.8651	2.2976	-4.7734
H	-1.1807	1.0836	-6.0026
H	-0.035	2.3995	-6.3326
H	-0.4675	-6.7482	8.5319

Conformer 7: E = -1379.09899411 Hartree

C	-2.257	-3.0902	0.5054
C	-2.3932	-2.3099	1.8451
C	-1.1251	-2.5086	2.7734
C	-2.5441	-0.7373	1.5629
C	-3.637	-2.8769	2.5761
C	-0.9102	-3.9502	3.4047
C	0.1508	-1.881	2.1494
C	-2.897	0.0593	2.8638
C	-1.2449	-0.2139	0.8841
C	-3.5753	-0.2985	0.4996
C	-3.4573	-4.3102	3.0841
C	-0.3478	-4.9676	2.3854
C	-2.2631	-4.4295	4.0513
C	0.1649	-3.825	4.5463
C	-0.0217	-0.4005	1.7707
C	-1.6004	1.2132	0.4178
C	-3.088	1.0785	0.0011
C	-2.187	-5.8232	4.7825
C	-0.072	-4.6999	5.7752
C	-0.6771	1.8737	-0.6507
C	-3.1922	-5.8483	5.9565
C	-2.5184	-7.0258	3.8747
C	-0.7846	-5.9829	5.4033
C	-0.4656	0.9955	-1.9073
C	-1.217	3.2675	-1.0149
O	0.614	2.0774	-0.0572
N	-0.3129	-7.1788	5.6087
C	0.3985	1.6424	-2.9996
C	0.6016	0.701	-4.159
C	1.7045	-0.015	-4.4538
C	1.7491	-0.8881	-5.6811
C	2.9615	-0.0438	-3.6276
O	0.9583	-7.1568	6.1865
H	-1.3525	-2.8409	-0.0524
H	-2.2423	-4.169	0.6382
H	-3.1141	-2.9174	-0.1511
H	-1.3256	-1.8956	3.6612
H	-3.8834	-2.2708	3.453
H	-4.5132	-2.8341	1.9172
H	0.9946	-1.9508	2.844
H	0.448	-2.433	1.2516
H	-3.8836	-0.2214	3.2453
H	-2.173	-0.0776	3.6686
H	-2.9599	1.1391	2.6997
H	-1.0688	-0.8012	-0.0224
H	-4.5973	-0.2472	0.8897
H	-3.5967	-0.9665	-0.3648
H	-4.3806	-4.5946	3.6028
H	-3.3593	-5.002	2.2433
H	0.4456	-4.5372	1.7687
H	0.1053	-5.8311	2.8841
H	-1.1078	-5.3714	1.7185
H	-2.4707	-3.6893	4.8418
H	1.1621	-4.044	4.1415
H	0.2254	-2.7917	4.9116
H	-0.0965	0.2103	2.677
H	0.8827	-0.0732	1.2475
H	-1.5512	1.8652	1.301
H	-3.2429	1.1522	-1.0794
H	-3.6751	1.8816	0.4619
H	-0.7126	-4.1671	6.4884
H	0.8756	-4.8718	6.2913
H	-3.1125	-6.7822	6.5256
H	-4.2255	-5.7613	5.6052
H	-3.0079	-5.0309	6.6627
H	-3.5306	-6.9598	3.4629
H	-1.818	-7.1259	3.0428
H	-2.4763	-7.9694	4.4326
H	-1.4326	0.713	-2.3395
H	0.0412	0.0679	-1.6132
H	-0.4682	3.8589	-1.5532
H	-1.4494	3.842	-0.1103
H	-2.1226	3.2103	-1.6263
H	0.4974	2.6387	0.7291
H	1.3527	1.9822	-2.5865
H	-0.0966	2.5372	-3.3943
H	-0.2586	0.6262	-4.8245
H	1.907	-1.9338	-5.3975
H	0.8218	-0.8373	-6.2615
H	2.5688	-0.577	-6.3369
H	3.7881	0.4092	-4.1844
H	2.867	0.4831	-2.6754
H	3.2316	-1.0787	-3.391
H	1.1681	-8.1061	6.2716

Conformer 8: E = -1379.09887102 Hartree

C	-1.7634	-3.5806	0.6439
C	-2.1537	-2.6702	1.8436
C	-1.0663	-2.7107	2.9937
C	-2.2957	-1.1481	1.3619
C	-3.491	-3.2093	2.4122
C	-0.9176	-4.0706	3.7981
C	0.273	-2.0685	2.544
C	-2.8854	-0.2319	2.4891
C	-0.9155	-0.6373	0.8466
C	-3.1468	-0.8808	0.1081
C	-3.3656	-4.5728	3.0989

C	-0.1581	-5.1532	2.9981
C	-2.3472	-4.5462	4.2561
C	-0.0594	-3.7839	5.0847
C	0.1033	-0.6438	1.9855
C	-1.2205	0.7017	0.1242
C	-2.6829	0.4986	-0.3842
C	-2.3561	-5.8586	5.1281
C	-0.4728	-4.5509	6.3389
C	-0.2548	1.1242	-1.038
C	-3.545	-5.8171	6.115
C	-2.4936	-7.1564	4.3054
C	-1.0751	-5.8944	5.9859
C	-0.6957	2.5214	-1.5712
C	1.2105	1.1391	-0.5927
O	-0.3755	0.1447	-2.0753
N	-0.6109	-7.0426	6.3872
C	0.1772	3.2078	-2.6381
C	0.1889	2.5059	-3.9679
C	1.2507	2.0916	-4.6872
C	1.0506	1.392	-6.0087
C	2.6943	2.2662	-4.3017
O	0.5427	-6.9081	7.1627
H	-0.7719	-3.3641	0.2417
H	-1.7665	-4.6394	0.8903
H	-2.4834	-3.5002	-0.175
H	-1.4444	-2.0314	3.7675
H	-3.8997	-2.5208	3.1589
H	-4.2402	-3.276	1.6136
H	0.975	-2.0155	3.3828
H	0.7562	-2.6848	1.7788
H	-3.9439	-0.4543	2.6611
H	-2.3707	-0.3221	3.4464
H	-2.8446	0.8322	2.2382
H	-0.5368	-1.3148	0.072
H	-4.2238	-0.8997	0.3042
H	-2.9501	-1.5972	-0.6936
H	-4.3559	-4.839	3.4871
H	-3.1131	-5.3437	2.3661
H	0.7109	-4.7444	2.4756
H	0.2353	-5.9386	3.6525
H	-0.7818	-5.6601	2.2637
H	-2.7064	-3.7438	4.9217
H	0.9996	-3.9874	4.8764
H	-0.0995	-2.7193	5.349
H	-0.1993	0.0378	2.7881
H	1.0822	-0.2937	1.6527
H	-1.2138	1.5074	0.8707
H	-2.7853	0.5476	-1.4728
H	-3.3246	1.2881	0.0248
H	-1.2396	-3.9839	6.8808
H	0.3795	-4.6299	7.0179
H	-3.5359	-6.6876	6.782
H	-4.5057	-5.8119	5.59
H	-3.5072	-4.9283	6.7548
H	-3.4229	-7.1767	3.7269
H	-1.6595	-7.3033	3.6159
H	-2.5192	-8.0394	4.9557
H	-0.7564	3.2115	-0.7187
H	-1.7093	2.4506	-1.9849
H	1.8749	1.4764	-1.3935
H	1.5624	0.128	-0.3665
H	1.3631	1.7858	0.2765
H	0.2759	0.3564	-2.7667
H	1.1789	3.3844	-2.2414
H	-0.2431	4.2072	-2.813
H	-0.8074	2.3302	-4.3777
H	1.4967	0.3925	-5.9783
H	-0.0084	1.275	-6.262
H	1.5244	1.9615	-6.8149
H	3.2322	2.7998	-5.0926
H	2.8359	2.8331	-3.3799
H	3.166	1.2876	-4.1642
H	0.7619	-7.8345	7.3777

Conformer 9: E = -1379.09863435 Hartree

C	-2.1734	-3.9662	0.5699
C	-2.1004	-2.9561	1.7515
C	-0.835	-3.216	2.6731
C	-2.0038	-1.4565	1.197
C	-3.3833	-3.1611	2.5961
C	-0.8421	-4.5765	3.5026
C	0.4943	-2.8912	1.9388
C	-2.134	-0.4004	2.3482
C	-0.682	-1.2894	0.3893
C	-3.0144	-1.0357	0.1149
C	-3.4189	-4.4767	3.3844
C	-0.6176	-5.8059	2.5925
C	-2.1943	-4.6162	4.3158
C	0.3182	-4.5339	4.5442
C	0.5206	-1.4863	1.3094
C	-0.8295	0.0614	-0.3594
C	-2.3723	0.1887	-0.5548
C	-2.3258	-5.7489	5.3967
C	0.2833	-5.6474	5.586
C	-0.0542	0.2326	-1.7109
C	-3.5801	-5.4873	6.2742
C	-2.474	-7.1703	4.8328
C	-1.0612	-5.7043	6.2898
C	-0.2209	1.6972	-2.2058
C	1.4304	-0.1272	-1.5896
O	-0.6204	-0.6296	-2.7054
N	-1.1987	-5.7809	7.5834
C	0.3686	1.9901	-3.5924
C	0.0493	3.3975	-4.0279
C	0.868	4.468	-4.0286
C	0.3856	5.8026	-4.5362
C	2.2893	4.4711	-3.536
O	0.0241	-5.7776	8.2576
H	-1.2548	-4.0064	-0.019
H	-2.3772	-4.9854	0.8925
H	-2.9986	-3.7377	-0.1097
H	-0.8857	-2.4596	3.4661
H	-3.4899	-2.3593	3.3337
H	-4.2712	-3.1027	1.9541
H	1.338	-2.9468	2.6337
H	0.6921	-3.6263	1.1525
H	-3.1612	-0.3563	2.7259
H	-1.4796	-0.5927	3.1991
H	-1.8954	0.6164	2.0226
H	-0.6251	-2.0624	-0.3846
H	-4.0064	-0.8041	0.5161
H	-3.1459	-1.7982	-0.657
H	-4.3454	-4.4595	3.9663
H	-3.5092	-5.3335	2.7118
H	-1.5509	-6.265	2.2627
H	-0.035	-5.5613	1.7012
H	-0.0524	-6.6007	3.0853

H	-2.1882	-3.6871	4.9117
H	1.2878	-4.587	4.0354
H	0.2946	-3.5724	5.0748
H	0.5408	-0.7261	2.0982
H	1.4627	-1.3763	0.7682
H	-0.492	0.8625	0.3123
H	-2.6843	0.2382	-1.6027
H	-2.7251	1.1128	-0.0815
H	1.0997	-5.4692	6.2913
H	0.4756	-6.6282	5.1423
H	-3.6362	-6.1832	7.1201
H	-4.5098	-5.6211	5.7127
H	-3.571	-4.4726	6.6881
H	-3.2954	-7.2367	4.1126
H	-1.5662	-7.5378	4.3524
H	-2.6884	-7.884	5.6382
H	0.226	2.38	-1.4707
H	-1.2892	1.9441	-2.251
H	1.9609	0.017	-2.536
H	1.5623	-1.191	-1.3688
H	1.9288	0.4691	-0.8194
H	-0.4655	-1.5505	-2.4354
H	-0.0554	1.3057	-4.3368
H	1.4474	1.819	-3.6047
H	-0.9699	3.5227	-4.3947
H	-0.6489	5.7627	-4.8931
H	0.4337	6.5518	-3.7392
H	1.0115	6.1389	-5.3693
H	2.4294	5.2686	-2.7983
H	2.9761	4.6485	-4.3698
H	2.5819	3.5371	-3.0508
H	-0.2496	-5.8379	9.1924

Conformer 10: E = -1379.09811452 Hartree

C	-2.3707	-4.0025	0.5572
C	-2.1531	-2.966	1.6975
C	-0.8509	-3.2867	2.5448
C	-1.991	-1.4932	1.0886
C	-3.3896	-3.057	2.6271
C	-0.8968	-4.6171	3.4212
C	0.4448	-3.0831	1.7138
C	-1.9719	-0.3973	2.2091
C	-0.7176	-1.442	0.1925
C	-3.0418	-1.0342	0.0619
C	-3.4627	-4.3431	3.4596
C	-0.8152	-5.8874	2.5437
C	-2.1931	-4.5376	4.318
C	0.3283	-4.6226	4.3864
C	0.529	-1.7051	1.0328
C	-0.8219	-0.1	-0.5795
C	-2.3611	0.108	-0.7087
C	-2.3307	-5.6242	5.4444
C	0.2865	-5.6987	5.4665
C	-0.1048	-0.0254	-1.9632
C	-3.5041	-5.2472	6.3894
C	-2.6128	-7.0482	4.9415
C	-1.0106	-5.6426	6.2543
C	-0.3286	1.3347	-2.6797
C	1.3969	-0.3211	-1.8677
O	-0.6693	-1.0398	-2.8122
N	-1.069	-5.6753	7.5557
C	0.1527	2.5667	-1.9084
C	-0.039	3.8196	-2.7209
C	0.9031	4.5404	-3.3603
C	0.5331	5.7982	-4.1041
C	2.3659	4.1947	-3.4225
O	0.1923	-5.7394	8.1514
H	-1.4998	-4.1182	-0.0912
H	-2.6139	-4.997	0.9261
H	-3.2256	-3.7413	-0.072
H	-0.796	-2.5036	3.3112
H	-3.3942	-2.2293	3.3433
H	-4.3112	-2.9555	2.0404
H	1.3279	-3.179	2.3531
H	0.5361	-3.8549	0.9432
H	-2.9601	-0.2864	2.6678
H	-1.2577	-0.5979	3.0087
H	-1.7139	0.5956	1.8294
H	-0.7673	-2.233	-0.565
H	-3.9812	-0.7091	0.5209
H	-3.2894	-1.8136	-0.6631
H	-4.3471	-4.2456	4.0964
H	-3.6522	-5.211	2.8229
H	-0.2756	-5.713	1.6099
H	-0.2752	-6.7039	3.029
H	-1.7967	-6.2902	2.2887
H	-2.0861	-3.5931	4.8789
H	1.2575	-4.7578	3.8206
H	0.4031	-3.6461	4.8836
H	0.6652	-0.9241	1.789
H	1.4331	-1.6889	0.4208
H	-0.4089	0.6906	0.0585
H	-2.7204	0.1082	-1.7424
H	-2.6367	1.0787	-0.2797
H	1.1565	-5.5549	6.1131
H	0.3846	-6.7035	5.0463
H	-3.5529	-5.9109	7.2611
H	-4.4755	-5.3317	5.8926
H	-3.3979	-4.2234	6.7658
H	-3.482	-7.0784	4.2772
H	-1.7657	-7.4938	4.4184
H	-2.823	-7.7197	5.7835
H	-1.3906	1.452	-2.9319
H	0.1736	1.3009	-3.6576
H	1.9018	-0.0856	-2.8123
H	1.5807	-1.3877	-1.7043
H	1.8803	0.256	-1.0739
H	-0.2221	-0.9897	-3.6752
H	1.1943	2.4499	-1.599
H	-0.4257	2.6855	-0.9852
H	-1.0726	4.1651	-2.7632
H	0.7816	5.6984	-5.1657
H	-0.536	6.0245	-4.0327
H	1.0815	6.6538	-3.6964
H	2.953	4.9446	-2.8827
H	2.5983	3.215	-2.999
H	2.7037	4.1771	-4.4644
H	-0.0238	-5.7566	9.103

Conformer 11: E = -1379.09782408 Hartree

C	-3.0198	-3.5393	0.8014
C	-2.3758	-2.4895	1.7526
C	-0.9746	-2.9763	2.3055
C	-2.1373	-1.1062	0.9754
C	-3.343	-2.3066	2.9501
C	-0.9826	-4.1943	3.3243

C	0.0827	-3.0844	1.1752
C	-1.6908	0.0391	1.9464
C	-1.1149	-1.331	-0.1796
C	-3.3191	-0.5426	0.1595
C	-3.4287	-3.5287	3.869
C	-1.2036	-5.5543	2.6231
C	-2.0513	-3.9118	4.4452
C	0.4346	-4.2695	4.0028
C	0.234	-1.7892	0.3589
C	-1.1863	-0.0393	-1.0232
C	-2.6758	0.3967	-0.8762
C	-2.1278	-5.0086	5.574
C	0.4407	-4.6701	5.4765
C	-0.7092	-0.0959	-2.5053
C	-2.5485	-4.351	6.9081
C	-3.1401	-6.1361	5.2842
C	-0.7175	-5.5906	5.7979
C	0.8219	-0.2572	-2.702
C	-1.4006	-1.2083	-3.3146
O	-1.1068	1.1301	-3.1444
N	-0.5958	-6.7953	6.2762
C	1.7581	0.6551	-1.8898
C	1.6643	2.1127	-2.2475
C	1.4158	3.1596	-1.4358
C	1.3525	4.5593	-1.9949
C	1.1902	3.0857	0.0499
O	0.7396	-7.1727	6.4329
H	-2.3756	-3.8208	-0.0336
H	-3.2864	-4.4657	1.3038
H	-3.9673	-3.182	0.3889
H	-0.6166	-2.153	2.9361
H	-3.027	-1.4695	3.5803
H	-4.3478	-2.0561	2.5881
H	1.0655	-3.3334	1.5892
H	-0.1738	-3.897	0.4876
H	-2.498	0.3097	2.6346
H	-0.8121	-0.2105	2.5428
H	-1.4382	0.9676	1.4263
H	-1.4842	-2.1362	-0.8226
H	-4.057	-0.0195	0.7766
H	-3.8535	-1.3182	-0.3947
H	-4.1133	-3.2784	4.6882
H	-3.8864	-4.3693	3.3406
H	-0.6268	-5.6393	1.6983
H	-0.8765	-6.3906	3.2503
H	-2.2479	-5.7492	2.3849
H	-1.6947	-2.9953	4.9441
H	1.0765	-4.9634	3.4435
H	0.9446	-3.2993	3.9459
H	0.6949	-1.0118	0.9778
H	0.9299	-1.9888	-0.4592
H	-0.5989	0.7415	-0.529
H	-3.2406	0.3626	-1.8124
H	-2.7196	1.4352	-0.5258
H	0.3279	-3.7758	6.1014
H	1.4117	-5.0986	5.7361
H	-2.5429	-5.0799	7.7274
H	-3.5558	-3.9264	6.849
H	-1.8616	-3.547	7.1957
H	-4.1589	-5.7514	5.1724
H	-2.8885	-6.7011	4.3841
H	-3.1755	-6.8607	6.107
H	1.0518	-0.0995	-3.7657
H	1.1059	-1.2945	-2.49
H	-1.1569	-1.1158	-4.3802
H	-2.4911	-1.1425	-3.2522
H	-1.0952	-2.2073	-2.9891
H	-0.7667	1.8678	-2.6075
H	2.7892	0.3425	-2.1017
H	1.6176	0.4772	-0.8229
H	1.8291	2.3132	-3.3077
H	1.5151	4.5832	-3.0775
H	0.3702	5.0009	-1.7974
H	2.1181	5.1887	-1.5296
H	0.176	3.4207	0.2915
H	1.3132	2.0829	0.4629
H	1.9012	3.736	0.5707
H	0.6628	-8.0779	6.7896

Conformer 12: E = -1379.09776823 Hartree

C	-3.7271	-2.4897	1.3475
C	-2.5812	-1.9719	2.2645
C	-1.2823	-2.8763	2.153
C	-2.1736	-0.4761	1.8582
C	-3.1108	-2.0342	3.7197
C	-1.4103	-4.358	2.7269
C	-0.6157	-2.7623	0.7554
C	-1.173	0.1539	2.8861
C	-1.6065	-0.4718	0.4075
C	-3.3163	0.5451	1.685
C	-3.2645	-3.4546	4.2776
C	-2.2865	-5.2528	1.8202
C	-1.9347	-4.2379	4.2105
C	0.0129	-4.9926	2.7943
C	-0.3398	-1.3121	0.3204
C	-1.5429	1.0206	0.0079
C	-2.7156	1.6603	0.8137
C	-1.9124	-5.5541	5.0682
C	0.0904	-6.3093	3.5604
C	-1.5878	1.3756	-1.5096
C	-2.2002	-5.2099	6.555
C	-2.9443	-6.6149	4.6558
C	-0.4982	-6.1751	4.9538
C	-0.3368	0.949	-2.3157
C	-2.8311	0.816	-2.2258
O	-1.6672	2.8117	-1.5858
N	0.0895	-6.6095	6.0327
C	0.9965	1.4785	-1.771
C	2.13	1.133	-2.7021
C	3.1465	0.2717	-2.4988
C	4.2019	0.0724	-3.5574
C	3.3532	-0.5634	-1.2652
O	1.3339	-7.1879	5.7743
H	-3.4245	-2.6087	0.3052
H	-4.124	-3.4522	1.6641
H	-4.5969	-1.8279	1.3715
H	-0.5478	-2.4247	2.8313
H	-2.4304	-1.5123	4.3998
H	-4.0731	-1.5124	3.7938
H	0.3443	-3.2877	0.7449
H	-1.2366	-3.2391	-0.0094
H	-0.3029	-0.4711	3.0907
H	-0.7698	1.1156	2.5562
H	-1.6674	0.3591	3.8414
H	-2.3384	-0.9369	-0.2598
H	-3.6942	0.9337	2.6363

H	-4.1695	0.1325	1.1409
H	-3.5934	-3.3419	5.3149
H	-4.0706	-3.9897	3.7692
H	-2.2644	-4.9337	0.7756
H	-1.9522	-6.2929	1.8007
H	-3.3308	-5.2819	2.1353
H	-1.1993	-3.5866	4.7137
H	0.396	-5.1752	1.7835
H	0.7045	-4.2833	3.2685
H	0.458	-0.8844	0.9373
H	0.0431	-1.3379	-0.7018
H	-0.6268	1.4645	0.4163
H	-3.492	2.1126	0.1903
H	-2.3253	2.466	1.4477
H	1.1404	-6.6129	3.5947
H	-0.4406	-7.1141	3.0444
H	-2.068	-6.0822	7.2067
H	-3.2305	-4.8744	6.7076
H	-1.5256	-4.4268	6.9194
H	-3.9575	-6.2026	4.621
H	-2.7266	-7.0719	3.6895
H	-2.9543	-7.4431	5.3756
H	-0.4463	1.3287	-3.3423
H	-0.2977	-0.1416	-2.4073
H	-2.8961	1.2071	-3.2485
H	-3.7602	1.1186	-1.7345
H	-2.812	-0.2759	-2.2907
H	-1.7479	3.0599	-2.5235
H	1.1805	1.0961	-0.7658
H	0.9583	2.5706	-1.6814
H	2.0994	1.6763	-3.6474
H	4.0265	0.6926	-4.4428
H	5.1893	0.3312	-3.161
H	4.2197	-0.973	-3.8826
H	4.3076	-0.3068	-0.7936
H	2.5723	-0.4357	-0.5141
H	3.3749	-1.6257	-1.5309
H	1.6324	-7.4542	6.6645

Conformer 13: E = -1379.09762826 Hartree

C	-1.9522	-3.6593	0.3937
C	-2.1016	-2.8005	1.6837
C	-0.8798	-3.0133	2.6744
C	-2.1654	-1.2408	1.3159
C	-3.404	-3.2688	2.381
C	-0.7723	-4.4524	3.3536
C	0.4482	-2.4623	2.0842
C	-2.5208	-0.3605	2.5611
C	-0.8203	-0.8179	0.6599
C	-3.138	-0.8112	0.1953
C	-3.32	-4.6638	3.0118
C	-0.3199	-5.5335	2.3445
C	-2.1644	-4.7532	4.0328
C	0.2898	-4.3875	4.4941
C	0.3553	-1.0029	1.6077
C	-1.0888	0.5955	0.1007
C	-2.5686	0.5128	-0.3547
C	-2.2262	-6.0094	4.9745
C	0.3247	-5.6092	5.4065
C	-0.1039	1.1451	-0.9773
C	-3.5664	-6.0048	5.7594
C	-2.1438	-7.3644	4.2552
C	-1.0494	-5.9133	5.9768
C	0.1071	0.1689	-2.1608
C	-0.5625	2.5401	-1.4284
O	1.179	1.3177	-0.3524
N	-1.2722	-6.1575	7.2373
C	0.9999	0.6745	-3.3099
C	0.237	1.4226	-4.3735
C	0.5253	2.6188	-4.924
C	-0.3669	3.206	-5.9882
C	1.7105	3.4778	-4.5767
O	-0.1147	-6.0826	8.0147
H	-1.0034	-3.5012	-0.1229
H	-2.023	-4.7284	0.5842
H	-2.7582	-3.4663	-0.3189
H	-1.0794	-2.3596	3.533
H	-3.674	-2.5877	3.1934
H	-4.2415	-3.2431	1.6726
H	1.2468	-2.5054	2.8311
H	0.7808	-3.0769	1.2421
H	-1.8435	-0.505	3.4042
H	-2.504	0.7117	2.3448
H	-3.5393	-0.5582	2.9094
H	-0.6371	-1.4657	-0.203
H	-4.1688	-0.6917	0.5454
H	-3.1636	-1.5213	-0.6344
H	-4.2825	-4.8365	3.5025
H	-3.2381	-5.437	2.2437
H	0.2949	-5.1215	1.541
H	0.3023	-6.3063	2.8022
H	-1.1568	-6.0599	1.883
H	-2.3283	-3.9046	4.7192
H	1.2938	-4.2616	4.0722
H	0.0987	-3.5039	5.118
H	0.2715	-0.3343	2.4716
H	1.2943	-0.746	1.1066
H	-1.0305	1.2953	0.946
H	-2.6885	0.5402	-1.442
H	-3.1273	1.3657	0.0483
H	1.0542	-5.4134	6.1971
H	0.679	-6.501	4.882
H	-3.5942	-6.7943	6.5202
H	-4.4259	-6.1861	5.1068
H	-3.7215	-5.0505	6.2754
H	-1.1623	-7.5608	3.8219
H	-2.3224	-8.1863	4.9599
H	-2.8931	-7.4481	3.4619
H	-0.8557	-0.1669	-2.5623
H	0.6155	-0.724	-1.7743
H	0.2363	3.0714	-1.9548
H	-0.8037	3.1676	-0.562
H	-1.4468	2.4969	-2.071
H	1.0727	1.9543	0.3759
H	1.433	-0.2043	-3.8057
H	1.8431	1.2409	-2.9082
H	-0.6441	0.8936	-4.7386
H	-1.2215	2.561	-6.218
H	-0.7605	4.1737	-5.6601
H	0.1961	3.3547	-6.9154
H	1.3719	4.4407	-4.1797
H	2.3725	3.0311	-3.8327
H	2.3117	3.6672	-5.4723
H	-0.4472	-6.2859	8.9095

Conformer 14: E = -1379.09746482 Hartree

C	-0.9372	-4.0277	0.7239
C	-1.7555	-3.0695	1.6364
C	-1.034	-2.8146	3.0227
C	-1.947	-1.649	0.9191
C	-3.1199	-3.7517	1.9107
C	-0.9456	-4.0415	4.0256
C	0.2865	-2.0179	2.8483
C	-2.9372	-0.7256	1.7093
C	-0.5529	-0.9819	0.7127
C	-2.4404	-1.654	-0.5388
C	-3.0214	-4.9975	2.7965
C	0.1347	-5.0675	3.6144
C	-2.3751	-4.6877	4.1615
C	-0.5205	-3.4883	5.4353
C	0.1025	-0.7053	2.0648
C	-0.81	0.2077	-0.2489
C	-2.0343	-0.2771	-1.0865
C	-2.4614	-5.8859	5.1815
C	-1.1636	-4.1771	6.637
C	0.3739	0.6741	-1.1642
C	-3.8716	-5.929	5.8127
C	-2.205	-7.2683	4.5465
C	-1.4721	-5.6285	6.3362
C	-0.0558	1.9584	-1.9289
C	1.6653	0.925	-0.3788
O	0.6599	-0.3378	-2.1375
N	-0.9963	-6.6406	7.0027
C	0.9554	2.4724	-2.9632
C	0.4187	3.6792	-3.6905
C	-0.0545	3.7395	-4.9508
C	-0.5228	5.048	-5.5338
C	-0.1823	2.5662	-5.8837
O	-0.1271	-6.2449	8.0216
H	0.0926	-3.7009	0.5673
H	-0.8782	-5.042	1.1109
H	-1.4046	-4.1486	-0.257
H	-1.6938	-2.1268	3.5652
H	-3.8023	-3.0622	2.4181
H	-3.6037	-4.0207	0.9635
H	0.7151	-1.7653	3.824
H	1.0365	-2.6264	2.3326
H	-3.9651	-1.0954	1.6304

H	-2.702	-0.6312	2.77
H	-2.9625	0.2989	1.3265
H	0.1057	-1.6707	0.1729
H	-3.518	-1.8245	-0.6301
H	-1.9389	-2.4093	-1.1494
H	-4.0394	-5.3751	2.9494
H	-2.4819	-5.7917	2.2736
H	-0.193	-5.7419	2.825
H	1.0562	-4.583	3.2809
H	0.4265	-5.7069	4.4546
H	-3.0019	-3.8872	4.5886
H	0.5718	-3.5392	5.539
H	-0.7669	-2.4223	5.5246
H	-0.4933	0.0004	2.6541
H	1.0839	-0.2412	1.9483
H	-1.1155	1.0736	0.3542
H	-1.8342	-0.3554	-2.1598
H	-2.8586	0.4376	-0.9758
H	-2.1147	-3.6862	6.8767
H	-0.5283	-4.0488	7.5165
H	-3.9386	-6.7114	6.5782
H	-4.6433	-6.1318	5.0631
H	-4.1223	-4.9827	6.3051
H	-2.9295	-7.4963	3.7581
H	-1.202	-7.3527	4.1228
H	-2.2983	-8.0709	5.2884
H	-0.2717	2.7553	-1.2046
H	-0.9922	1.7623	-2.4664
H	2.474	1.272	-1.0297
H	2.0511	-0.0032	0.0542
H	1.5185	1.6638	0.4149
H	0.9892	-1.1252	-1.6723
H	1.2351	1.6763	-3.6587
H	1.8825	2.7839	-2.4696
H	0.4465	4.5972	-3.1031
H	-0.4091	5.8806	-4.8317
H	0.0546	5.2898	-6.4321
H	-1.5815	4.985	-5.8061
H	0.5026	2.6823	-6.7299
H	-1.2042	2.5058	-6.2735
H	0.0305	1.6069	-5.4066
H	0.1375	-7.0972	8.4165

Conformer 15: E = -1379.09730329 Hartree

C	-3.3431	-2.7507	1.0726
C	-2.5262	-2.1684	2.2624
C	-1.1391	-2.9161	2.448
C	-2.2172	-0.6154	2.0196
C	-3.3815	-2.3779	3.5374
C	-1.2226	-4.4363	2.9198
C	-0.1682	-2.6393	1.2681
C	-1.5764	0.0548	3.2831
C	-1.3227	-0.4598	0.7535
C	-3.399	0.285	1.6065
C	-3.4929	-3.8385	3.9924
C	-1.7378	-5.3609	1.7928
C	-2.1023	-4.4679	4.2299
C	0.2074	-4.9162	3.3165
C	0.0238	-1.1421	0.9639
C	-1.3529	1.052	0.426
C	-2.7413	1.5159	0.9633
C	-2.1304	-5.826	5.0195
C	0.2546	-6.2625	4.0319
C	-1.1007	1.4934	-1.0523
C	-2.811	-5.6112	6.3988
C	-2.8945	-6.9643	4.3261
C	-0.6662	-6.2804	5.2393
C	0.3637	1.2241	-1.4768
C	-2.076	0.845	-2.0504
O	-1.3153	2.9155	-1.0719
N	-0.3093	-6.7132	6.4155
C	0.8153	1.941	-2.7565
C	2.2387	1.5889	-3.0995
C	2.6696	0.7331	-4.0472
C	4.1441	0.5244	-4.2797
C	1.7808	-0.0915	-4.9377
O	1.0215	-7.1342	6.4551
H	-2.7808	-2.7787	0.137
H	-3.6924	-3.7656	1.2519
H	-4.262	-2.1857	0.8956
H	-0.6536	-2.4323	3.3045
H	-2.9549	-1.827	4.3816
H	-4.389	-1.9702	3.3873
H	0.8225	-3.0519	1.4831

H	-0.5172	-3.1339	0.3563
H	-0.7221	-0.4913	3.685
H	-1.2088	1.0667	3.09
H	-2.3116	0.1588	4.0881
H	-1.8048	-0.9648	-0.0891
H	-4.0435	0.5627	2.4468
H	-4.0348	-0.1782	0.8477
H	-4.0738	-3.8266	4.9194
H	-4.0818	-4.4254	3.283
H	-1.5012	-4.975	0.7985
H	-1.2856	-6.3553	1.8204
H	-2.8159	-5.523	1.8372
H	-1.595	-3.7744	4.9226
H	0.8435	-4.9918	2.4268
H	0.6726	-4.1677	3.972

H	0.5839	-0.6652	1.7757
H	0.6516	-1.0607	0.0745
H	-0.5961	1.5636	1.0375
H	-3.398	1.9404	0.1986
H	-2.5976	2.3036	1.713
H	1.2935	-6.4525	4.3152
H	-0.0347	-7.0858	3.3728
H	-2.7365	-6.5038	7.0318
H	-3.8804	-5.4004	6.3013
H	-2.3457	-4.7841	6.947
H	-3.911	-6.6632	4.0544
H	-2.3946	-7.3323	3.4292
H	-2.979	-7.8329	4.9913
H	0.5075	0.1478	-1.6188
H	1.034	1.5374	-0.6651

H	-2.0056	1.3136	-3.038
H	-3.1183	0.9721	-1.7435
H	-1.8834	-0.2249	-2.1741
H	-1.4382	3.1982	-1.9943
H	0.7684	3.0277	-2.6174
H	0.144	1.7155	-3.5888
H	2.9814	2.1176	-2.501
H	4.761	1.1439	-3.6203
H	4.4123	-0.5219	-4.0999
H	4.4036	0.7785	-5.3127
H	0.7238	-0.0338	-4.6677
H	1.8827	0.2351	-5.9776
H	2.0654	-1.1476	-4.8785
H	1.1256	-7.4195	7.3827
